# Mitochondrial Dynamics Participate in an Early Metabolic Adaptation of Glioblastoma Multiforme T98G Cells to Doxorubicin-Induced Chemotherapeutic Stress

**DOI:** 10.3390/cells15100899

**Published:** 2026-05-14

**Authors:** Maciej Pudełek, Maksym Pudełek, Julia Przeniosło, Sylwia Kędracka-Krok, Zbigniew Madeja, Jarosław Czyż

**Affiliations:** 1 Department of Cell Biology, Faculty of Biochemistry, Biophysics and Biotechnology, Jagiellonian University, Gronostajowa 7, 30-387 Krakow, Poland; maksym.pudelek@student.uj.edu.pl (M.P.); julia.przenioslo@jcj.pl (J.P.); z.madeja@uj.edu.pl (Z.M.); 2 Department of Physical Biochemistry, Faculty of Biochemistry, Biophysics and Biotechnology, Jagiellonian University, Gronostajowa 7, 30-387 Krakow, Poland; sylwia.kedracka-krok@uj.edu.pl

**Keywords:** glioblastoma, mitofusin 2, drug-retention, doxorubicin, mitochondria, metabolic stress, lysosomes

## Abstract

**Highlights:**

**What are the main findings?**

- Pulse DOX treatment enhances mitochondrial networking and ATP production in T98G cells, supporting the activity of ABC transporters and lysosomal V-ATPases, and facilitating extranuclear DOX sequestration;
- Redundancy through mitochondrial chaperones (including mitofusin (MFN)2) and ABC transporters allows partial compensation of functional MFN2/ABC deficiency in minute T98G sub-populations.

**What are the implications of the main findings?**

- A “resistance triad”, which coordinates metabolic T98G reprogramming, the activation of the drug-relocation and drug-retention axis, determines the recovery of GBM populations from chemotherapeutic stress;
- Redundancy of a “resistance triad” facilitates the evolution of T98G drug resistance under chemotherapeutic stress and stresses the need for simultaneous targeting of multiple GBM stress-resistance systems.

**Abstract:**

Chemotherapy-induced metabolic reprogramming of glioblastoma multiforme (GBM) cells increases intracellular levels of reductive and energetic carriers, thereby fueling drug-relocation and retention systems and enhancing GBM drug-resistance. We have previously shown the role of this process in the adaptation of poly(morpho)nuclear “giant” cells (PGCs) in T98G populations to doxorubicin (DOX)-induced stress. Here, we addressed the role of a “resistance triad”, which coordinates metabolic T98G reprogramming with the activation of the drug-relocation and drug-retention axis, in the recovery of GBM populations from chemotherapeutic stress. A combination of proteomic analyses with metabolic and phenotypic profiling of pulse DOX-treated T98G cells revealed the significance of mitochondrial dynamics for the efficiency of the T98G “resistance triad”. DOX-induced mobilization of ATP-generating systems and ATP-dependent anabolic pathways was accompanied by the formation of DOX-negative, “mosaic” mitochondrial networks and the upregulation of mitofusin-2 (MFN2) in T98G PGCs. Transient MFN2 down-regulation correlated with the respiratory capacity of T98G cells, while impairing cell welfare in the absence and presence of DOX. However, minute fractions of PGCs, which withstood combined MFN2 down-regulation and pulse DOX treatment, retained mitochondrial networks and displayed efficient ABC transporter-/V-type channel-dependent lysosomal DOX retention. Collectively, a “triad” of mitochondrial activation, ABC transporter-dependent perinuclear redistribution and V-type channel-mediated lysosomal DOX compartmentalization determines DOX resistance of T98G cells. Whereas MFN2-dependent mitochondrial rearrangements may contribute to these processes, complementary adaptative mechanisms can compensate MFN2 dysfunction, limiting its potential as a therapeutic target.

## 1. Introduction

Gliomas represent a heterogeneous group of neural neoplasms arising from glial progenitors, with high-grade glioblastoma multiforme (GBM; WHO grade IV glioma) exhibiting the most aggressive clinical behavior. The short median survival (less than 15 months) and poor five-year survival rate (<10%) of GBM patients subjected to combined treatment strategies result from the adaptive responses of GBM cells [1,2,3]. These responses drive the microevolution of GBM tumors toward invasive and drug-resistant phenotypes [4,5], facilitating aggressive tumor recurrence following cytostatic drug application [6,7,8,9,10,11,12,13]. Such findings underscore the urgent need to develop new therapeutic strategies for GBM [9,14,15].

The activation of drug-resistance systems increases the energetic demands of tumor cells under chemotherapeutic stress, necessitating compensatory metabolic adjustments [16,17]. This highlights the critical role of metabolic plasticity and reprogramming in GBM cell adaptation to chemotherapy-induced stress. Recently, pro-aerobic metabolic reprogramming of tumor cells has been identified as a key mechanism that facilitates the effective management of microenvironmental resources [18,19,20,21,22,23,24]. This adaptation compensates for stress-induced metabolic imbalance and provides the energetic support required for drug-resistance systems [5,24,25]. A notable example is the correlation between increased chemoresistance and pro-oxidative reprogramming of cancer cells [24,26,27]. It ensures a readily available energy supply for drug-efflux, drug-degradation, and repair systems. When coupled with enhanced metabolic adaptation of drug-resistant cells to microenvironmental limitations, i.e., their metabolic plasticity [18,28,29], pro-oxidative reprogramming enables tumor cell survival under adverse conditions [18,24,28,30,31]. The relationship between GBM metabolic plasticity, drug resistance, and invasiveness positions metabolic inhibitors as key tools to enhance the efficiency of GBM chemotherapy. Indeed, inhibition of mitochondrial oxidation has been shown to sensitize cancer cells to chemotherapy [23,32].

We have recently shown that poly(morpho)nuclear giant cells (PGCs) in GBM T98G populations withstand chemotherapeutic stress induced by doxorubicin (DOX) through its intracellular neutralization and sequestration [33]. Both processes attenuate DOX toxicity by reducing its bioavailability within critical cellular compartments. At the microenvironmental level, intracellular drug retention systems are crucial for the maintenance of low DOX bioavailability in the vicinity of cancer stem cells [34,35,36,37,38]. At the (sub)cellular level, the mobilization of ATP-binding cassette (ABC) transporters [38,39,40], DNA repair systems [41,42], autophagy [43,44,45], reactive oxygen species (ROS) scavengers and enzymatic drug-inactivators (P450 complex) in DOX-treated PGCs correlates with their aerobic activation [33]. Presumably, this supports their bioenergetic capacity, thereby sustaining long-term intracellular DOX retention. However, the links between the metabolic adaptation of T98G cells to increased metabolic burden from the DOX retention axis and mitochondrial dynamics have not been addressed. In this context, the function of mitofusins (MFN1 and 2) is an intriguing topic, as these proteins determine the balance between mitochondrial fusion and fission, concomitantly participating in aberrant ER-mitochondrial tethering in cancer cells [46,47,48].

Mitochondrial dynamics, particularly the processes of fusion and fission, play a central role in regulating mitochondrial bioenergetics and stress responses [49,50]. Fragmented mitochondria typically exhibit a decreased membrane potential, impaired oxidative phosphorylation (OXPHOS), and reduced ATP synthesis [50,51,52]. In turn, mitochondrial fusion enhances respiratory capacity, particularly in tumor cells exposed to chemotherapeutic stress [49,50,51,52,53]. This process promotes the homogenization of mitochondrial matrix contents, compensation of local damages, and the maintenance of the inner-membrane potential [51,52]. This evidence implies mitochondrial fusion events and the formation of interconnected mitochondrial networks in the metabolic plasticity of PGCs. Conceivably, these processes facilitate mitochondrial adaptation to DOX-induced chemotherapeutic stress and the associated metabolic burden. However, the mechanisms underlying mitochondrial dynamics in DOX-exposed PGCs, and their significance for the remarkable DOX resistance of these cells, remain poorly understood. To address this issue, (i) we analyzed the DOX-induced mitochondrial remodeling and its significance for the coordination of metabolic T98G reprogramming with the activation of the DOX-relocation and DOX-retention axis (i.e., the “resistance triad”). Next, (ii) we investigated mechanisms of DOX-induced mitochondrial remodeling in T98G cells and (iii) addressed the “resistance triad” redundancy as a determinant of T98G adaptation to DOX-induced stress.

## 2. Materials and Methods

### 2.1. Cell Culture

Human glioblastoma multiforme (GBM) T98G and U87-MG (ATCC, CRL-1690 and HTB-14, respectively; not listed as commonly misidentified cell lines by the International Cell Line Authentication Committee) cells were maintained under standard culture conditions in high-glucose (4500 mg/L) DMEM medium (Sigma-Aldrich, St. Louis, MO, USA; No. D6429; up to 25 passages), supplemented with 10% heat-inactivated fetal bovine serum (FBS; Gibco, Grand Island, NY, USA; No. A3840402) and 1% Antibiotic-Antimycotic Solution (Merck, No. A5955), as described previously [33,54]. The identity of the T98G line was confirmed by the International Cell Line Authentication Service (ICLAC; 2019). For each experiment, cells were collected using Ca^2+^/Mg^2+^-free DPBS/0.5 mM UlraPureTM EDTA solution (Invitrogen, Grand Island, NY, USA; No. 15575020;/Invitrogen, Grand Island, NY, USA; No. 14190144), counted on a Z2 particle counter (Beckman Coulter, Brea, CA, USA) and plated into multi-well tissue culture plates (Falcon^®^; Becton, Dickinson and Company, Franklin Lakes, NJ, USA). Unless stated otherwise, the cells were exposed to pulse DOX treatment for 48 h (1 µM; prepared from 1 mM Doxorubicin (DOX; Sigma-Aldrich, St. Louis, MO, USA; No. D1515) stock solution in sterile DMSO (Sigma-Aldrich, St. Louis, MO, USA; No. D8418)). Long-term effects of DOX were evaluated at the indicated time points after drug removal. Cells cultured with 0.1% DMSO served as control conditions. Where indicated, 1 µM Elacridar (ABCB1 inhibitor, Sigma-Aldrich, St. Louis, MO, USA; No. SML0486; from 100 µM stocks in DMSO), 10 nM bafilomycin A1 (V-ATPase inhibitor; Sigma-Aldrich, St. Louis, MO, USA; No. B1793), 5 µM Fumitremorgin C (ABCG2 inhibitor; Sigma-Aldrich, St. Louis, MO, USA; No. 344847 from 100 µM stocks in DMSO) and 10 µM BPTES (glutaminase inhibitor; Sigma-Aldrich, St. Louis, MO, USA; No. SML0601 from 10 mM stock in DMSO). No state or ethical approval was required for this study. No patient consent was required for this study.

### 2.2. Viability and Cytotoxicity Tests

To assess cell viability, cells were seeded into 12-well culture plates (Corning^®^Costar^®^, Corning, NY, USA) at an initial density of 2 × 10^4^ cells/well and subjected to a pulse DOX exposure protocol. At predetermined time points, cells were detached with TrypLE and, together with the collected culture medium, centrifuged (180 *g*; 5 min.). Cell pellets were then resuspended in fresh culture medium, and viability was assessed by trypan blue exclusion assay (1:1 *v*/*v*; Sigma-Aldrich, St. Louis, MO, USA; No. T8154) using a Bürker counting chamber (Marienfeld, Poly-Optik GmbH, Bad Blankenburg, Germany). An MTT assay was used to assess EC_50_ values 48 h after incubation, as previously described in [33], and changes in relative cellular metabolic activity as a determinant of DOX cytotoxicity. Cells were seeded into 96-well plates (Eppendorf AG, Hamurg, Germany) at a density of 5 × 10^3^ cells per well and exposed to DOX (1 µM) followed by the addition of thiazolyl blue tetrazolium bromide (Sigma-Aldrich, St. Louis, MO, USA; No. M5655) water solution to each well (1 mg/mL; 1:10) and incubation for 2 h at 37 °C. Subsequently, cells were subjected to dissolution with isopropanol and 570 nm absorbance measurement (MultiskanTM FC Microplate Reader; Thermo Fisher Scientific; Waltham, MA, USA). For morphometric analysis, cell areas were imaged using interference-modulation contrast (IMC; Hoffman contrast) and manually measured in ImageJ software (v. 1.54f). Intracellular DOX distribution/localization was visualized using a Leica Stellaris 5 confocal microscope (Leica Microsystems GmbH, Wetzlar, Germany; Core Facility of FBBB); operated via LAS X (v. 1.4.4.21655) software (objective Plan Apochromat 40×/1.3 N; oil immersion; laser 488 nm (20 mW)). DOX (co)localization with the lysosomal marker (LysoTracker Deep Red; Thermo Fisher Scientific, Waltham, MA, USA; No. L12492; working concentration 50 nM in FluoroBrite^TM^ DMEM) and DNA (Hoechst 33342) was quantified using a custom Python-based image analysis pipeline developed with the assistance of Claude Sonnet 4.6 Pro (Anthropic; https://claude.ai, accessed on 11 May 2026). Binary masks were generated for each channel using Otsu thresholding followed by morphological closing, and intensity line profiles were extracted along algorithmically optimized scan lines. The Pearson correlation coefficient (PCC) was calculated both along the scan line profiles and pixel-wise within the union mask, and supplemented by Manders’ colocalization coefficients (M1, M2) and the Intensity Correlation Quotient (ICQ).

### 2.3. LC-MS/MS Proteomic Analysis: Orbitrap Q Exactive

Cell preparation and LC-MS/MS analysis were performed as described previously [33]. Briefly, cells were lysed in 2% SDS/0.10 M Tris, pH 7.5, sonicated (Bioruptor UCD-200, Diagenode SA, Seraing, Belgium; high intensity, 30 s ON/30 s OFF, power 320 W), heated at 95 °C for 5 min and clarified by centrifugation (20,000× *g*, 10 min, RT). Proteins were processed by FASP, digested and analyzed by nanoLC-MS/MS on a Q-Exactive Orbitrap (UltiMate 3000RS nanoLC; Thermo Fisher Scientific, Waltham, MA, USA). MS settings: full MS *m*/*z* 300–2000, R = 70 000 (*m*/*z* 200); Top12 precursors (z ≥ 2) fragmented by HCD (NCE 27%, isolation 1.2 *m*/*z*); MS/MS acquired in the Orbitrap at R = 17 500 (*m*/*z* 200). Raw files were searched with MaxQuant/Andromeda against SwissProt (*H. sapiens*) + contaminants; FDR < 1% (peptide and protein). LFQ intensities were analyzed in Perseus (ANOVA with permutation FDR < 0.05, Tukey post hoc; n = 3 independent biological replicates). STRING v12.0 was used to construct interaction networks based on Gene Ontology annotations. All fold-change values presented in this study are statistically significant relative to the control condition.

### 2.4. LC-MS/MS Proteomic Analysis: Orbitrap Astral

#### 2.4.1. Sample Preparation and Measurement

Protein samples (20 µg) were prepared for LC-MS/MS analysis using the SP3 sample preparation protocol [55]. Resulting peptides were suspended in 0.1% formic acid (FA) and 250 ng of peptide mixture was analyzed with an Astral Orbitrap mass spectrometer coupled to a Vanquish Neo UHPLC (both Thermo Fisher Scientific, Waltham, MA, USA). UHPLC was operated in a direct injection mode. Peptides were separated using an Aurora Ultimate XT 25 cm × 75 µm C18 column (IonOpticks, Collingwood, VIC, Australia) in a 60 min gradient of acetonitrile (ACN) at a flow rate of 0.5 µL/min at 55 °C. The gradient consisted of 3 major steps: 2–10% B 10 min, 10–25% B 32 min, 25–45% B 18 min (buffer B: 80% ACN, 0.1% FA; buffer A: 0.1% FA). The eluting peptides were analyzed with an Orbitrap Astral mass spectrometer using the DIA method. Data were collected within 200 windows of 3 Th for the precursors from the mass range of 380–980 *m*/*z*. The maximum injection time (IT) was set to 5 ms and normalized AGC target to 500%. The isolated ions were fragmented with the normalized collision energy set to 25%. Astral scan range was 150–2000 *m*/*z*. Additionally, full-MS spectra were acquired every 0.6 s with a resolution of 240,000, a scan range of 380–980 *m*/*z*, a maximum IT of 3 ms and a normalized AGC target of 500%.

#### 2.4.2. Data Analysis

Raw files were analyzed in DIA-NN 2.2.0 [56] using an in silico DIA-NN-predicted spectral library generated from human reference proteome (UniProtKB, 20 663 sequences, downloaded in September 2025). The DIA-NN spectral library building, as well as the search, was performed using the following settings: protease—Trypsin/P, missed cleavages—up to 1, maximum number of variable modifications—2, modifications—C carbamidomethylation, N-term M excision, M oxidation, protein N-terminal acetylation, peptide length range—7–30, precursor charge range—2–4, precursor *m*/*z* range—380–980, fragment ion *m*/*z* range—150–2000, mass accuracy—10 ppm, MS1 accuracy—4 ppm, scan window—7, scoring—peptidoforms, proteotypicity—genes, machine learning—NNs (cross-validated), quantification strategy—QuantUMS (high precision), cross-run normalization—RT-dependent, library generation—IDs, RT and IM profiling, speed and RAM usage—optimal results. MBR and protein inference were enabled. The resulting pg_matrix was filtered for protein groups that satisfy the Global.PG.Q.Value threshold of 0.01. The list of protein groups that meet this criterion was obtained by filtering the main output file in RStudio (v2024.12.1+563). Then, non-human cRAP proteins were excluded. The data matrix was further processed in Perseus (v2.1.5.0) [57]. Protein intensity values were log2 transformed and the matrix was filtered for proteins that had at least 3 valid values in at least one group. Then, the remaining missing values were imputed by a constant low value. In order to reveal changes between the groups, Student’s *t*-test with Benjamini–Hochberg FDR set to 0.05 was used (n = 3 independent biological replicates). Proteins that satisfied the threshold of q-value < 0.05 and |Fold change| > 1.2 were considered differential. All fold-change values presented in this study are statistically significant relative to the control condition.

### 2.5. Immunocytochemistry, Fluorescence Microscopy and Fluorimetry

For immunocytochemical analyses, cells were plated onto coverslips placed in 12-well culture plates at a density of 2 × 10^4^ cells/well. At 24 h after seeding, cells were subjected to a pulse DOX exposure protocol followed by a recovery phase. At selected time points, samples were fixed with 3.7% (*v*/*v*) formaldehyde and subsequently permeabilized with 0.1% (*v*/*v*) Triton X-100. Non-specific binding sites were blocked with 2% (*w*/*v*) BSA (30 min, 37 °C). Specimens were incubated with the appropriate primary antibodies (in 2% BSA/0.01% Tween) for 45 min at room temperature. Preparations were then washed twice in 2% BSA and incubated for a further 45 min with the corresponding secondary antibodies and/or Hoechst 333258 (in 2% BSA/0.01% Tween). In particular, immunolocalization of specific proteins was performed with polyclonal rabbit anti-ABCB1 (Sigma-Aldrich, St. Louis, MO, USA; No. HPA002199; 1:250), polyclonal rabbit anti-ABCC1 (Sigma-Aldrich, St. Louis, MO, USA; No. HPA002380; 1:250) and monoclonal rabbit anti-ABCG2 (Sigma-Aldrich, St. Louis, MO, USA; No. ZRB1217; 1:100), rabbit anti-MFN1 (Cell Signaling Technology, Danvers, MA, USA; No. 14739; 1:100), rabbit anti-MFN2 (Cell Signaling Technology, Danvers, MA, USA; No. 11925; 1:100), rabbit anti-MFF (Cell Signaling Technology, Danvers, MA, USA; No. 84580; 1:100) and anti-GLS (glutaminase; ABclonal, Woburn, MA, USA; No. A11043; 1:100). After washing with 2% BSA, the 2% BSA/0.01% Tween-supplemented cocktails of the following secondary antibodies were applied for 45 min.: AlexaFluor488-conjugated chicken anti-rabbit IgG (Invitrogen, Grand Island, NY, USA; No. A21441), AlexaFluor647-conjugated chicken anti-rabbit IgG (Invitrogen, Grand Island, NY, USA; No. A21443), AlexaFluor546-conjugated phalloidin (Invitrogen, Grand Island, NY, USA; No. A22283; for F-actin visualization) and Hoechst 33258 (Sigma-Aldrich, St. Louis, MO, USA; for DNA staining; 1 µg/mL). Final washes were performed in distilled water and coverslips were mounted onto microscope slides using Dako Mounting Medium (Agilent, Santa Clara, CA, USA; No. CS703). Microphotographs were acquired either with a Leica DMI6000B fluorescence microscope or a Leica Stellaris 5 confocal microscope (Leica Microsystems GmbH, Wetzlar, Germany; Core Facility of FBBB); both operated via LAS X software (objectives: Plan Apochromat 40×/1.3 NA and 63×/1.4 NA; oil immersion) [33]. For confocal imaging, lasers at 405 nm (50 mW), 488 nm (20 mW), 561 nm (20 mW) and 638 nm (30 mW) were used according to the excitation/emission characteristics of the fluorophores. Signals were collected with point spectral Power HyD S detectors. For quantitative fluorescence analysis, image stacks (fluorescence microscopy) were acquired in the green channel (filter set GFP; excitation 455–495, emission 505–555), red channel (filter set N2.1; excitation BP515–560, emission 590LP) and blue channel (filter set A4; excitation BP360/40, emission BP470/40) using widefield fluorescence microscopy (Leica DMI 6000B fluorescence microscope; Leica Microsystems GmbH, Wetzlar, Germany). Quantitative confocal imaging was performed using laser lines at 405 nm (50 mW), 488 nm (20 mW), 561 nm (20 mW), and 638 nm (30 mW) for the blue, green, red, and far-red channels, respectively. To ensure inter-experimental comparability, all image stacks were acquired under identical illumination conditions, including consistent laser power, camera gain, and exposure time. Raw images were processed in ImageJ (v. 1.54f) software by background subtraction prior to fluorescence intensity quantification. Mean fluorescence intensity values for each target protein (i.e., MFN1, MFN2, and MFF) were determined from a minimum of 50 individual cells per experimental condition in 3 independent replicates (n = 3).

### 2.6. Visualization of Mitochondrial Architecture and Membrane Potential

Mitochondrial architecture was visualized using CellROX Deep Red or MitoTracker Green (Invitrogen, Grand Island, NY, USA; No. C10422 and M7514, respectively). Cells were cultured on glass-bottom dishes (Thermo Fisher, Waltham, MA, USA) at a density of 2 × 10^4^ cells/dish and incubated with 2.5 µM CellROX Deep Red or 100 nM MitoTracker Green for 30 min. Both dyes were diluted in FluoroBrite™ DMEM medium. The staining solution was then replaced with FluoroBrite™ DMEM supplemented with 10% FBS and 1% GlutaMAX. Imaging was performed on a Leica Stellaris 5 microscope (Leica Microsystems GmbH, Wetzlar, Germany) equipped with environmental control (5% CO_2_, 37 °C). Acquired images were analyzed using ImageJ software (v. 1.54f). Mitochondrial membrane potentials (ΔΨm) were assessed using the cationic dye JC-1 (Thermo Fisher Scientific, Waltham, MA, USA; No. T3168), which exhibits potential-dependent accumulation in mitochondria, forming red-fluorescent aggregates at high ΔΨm and remaining in green-fluorescent monomeric form at low ΔΨm. Cells were incubated with 1 µM JC-1 in complete culture medium for 20 min at 37 °C, washed with phenol red-free FluoroBrite^®^ DMEM to remove excess dye and immediately subjected to live-cell imaging. Fluorescence imaging was performed using a Leica DMI 6000B fluorescence microscope (Leica Microsystems GmbH, Wetzlar, Germany) equipped with appropriate filter sets (green channel: Ex ~488 nm/Em ~530 nm for JC-1 monomers; red channel: Ex ~540–561 nm/Em ~590 nm for JC-1 aggregates). Imaging settings (gain, exposure time) were kept constant across all experimental conditions. Mitochondrial membrane potential distribution was analyzed using a custom Python pipeline developed with the assistance of Claude Sonnet 4.6 Pro (Anthropic; https://claude.ai). Individual mitochondria were segmented from confocal JC-1 fluorescence images via combined red/green channel thresholding and connected-component labeling, with the principal axis of each object determined by principal component analysis (PCA). Fluorescence intensity profiles of both channels (green monomer—low Δψ; red aggregate—high Δψ) were sampled along the major axis using bilinear interpolation and normalized to their respective maxima, enabling visualization of spatial membrane potential distribution within and between individual mitochondria. Mitochondrial morphometry was performed using a custom Python pipeline based on the Frangi tubeness filter sigmas 1–4) applied to fluorescence images, with a global Otsu threshold calculated across all experimental conditions to eliminate inter-condition intensity bias. Segmented objects were filtered to 1–20 µm (to exclude noise and fused networks) and measured via skeletonization. Cells exhibiting a predominance of fused (long tubular structures) or hyperfused (networks) mitochondria were scored manually by visual inspection.

### 2.7. Transmission Electron Microscopy (TEM)

Ultrastructural identification of mitochondria in the DOX-exposed cells was performed with transmission electron microscopy (TEM). Cells were seeded on UVC-sterilized Thermanox^®^ (Invitrogen, Grand Island, NY, USA; No. 174942) membranes or glass slides dedicated to TEM in 12-well cell culture plates (Corning^®^Costar^®^, Corning, NY, USA). Cells were fixed with 2.5% glutaraldehyde and 2% paraformaldehyde for 1 h at 4 °C. After fixation, cells were rinsed three times for 10 min in 0.1 M cacodylate buffer. Specimens were then post-fixed in 1% osmium tetroxide for 30 min at room temperature, rinsed three times for 10 min in distilled water, and stained with 2% aqueous uranyl acetate for 30 min. Dehydration was carried out through a graded acetone series (25%, 50%, 70%, 90%, 96% and 2 × 100%; 10 min per step). Samples were subsequently infiltrated with mixtures of pure acetone and Epon resin (ratios 1:1, 1:2 and 2:1; 30 min for each mixture), followed by three exchanges in pure Epon resin (incubations of 1 h, overnight and 1 h). After polymerization of the resin at 60 °C and removal of the coverslips, 60 nm sections were collected onto formvar-coated TEM grids. Observations were performed with a transmission electron microscope (TEM) JEM-2100HT (Jeol Ltd., Tokyo, Japan; Transmission Electron Microscopy Laboratory; Institute of Zoology and Biomedical Research, Jagiellonian University, Kraków) or a JEM 1400 (Jeol Ltd., Tokyo, Japan; Laboratory of Electron Microscopy; Nencki Institute of Experimental Biology, Warsaw).

### 2.8. Immunoblotting

Visualization of protein levels was carried out using Western blot analysis. Cells were seeded in 25 cm^2^ Falcon^®^ culture dishes at a density of 5 × 10^4^ cells per dish and grown to 70–80% confluence, followed by pulse DOX treatment. At defined time points after treatment, cells were harvested directly using cold (~4 °C) Ca^2+^/Mg^2+^-free PBS/EDTA, pelleted by centrifugation, and lysed in buffer supplemented with a protease inhibitor cocktail, followed by freeze–thaw cycles and sonication. Total protein concentration was determined using the Bradford assay. Equal amounts of protein (20 µg) were separated by SDS–PAGE on 12% polyacrylamide gels according to the Laemmli protocol and subsequently transferred onto PVDF membranes (Immun-Blot^®^ PVDF Membrane, No. 1620177; Bio-Rad, Hercules, CA, USA [33,54]). Blocking of unspecific staining was performed with skimmed milk/TBST solution. For protein immunodetection, the following primary antibodies were used: monoclonal IgG rabbit anti-MFN-2 (Cell Signaling Technology, Danvers, MA, USA; No. 11925T; 1:1000) and monoclonal IgG rabbit anti-MFF (Cell Signaling Technology, Danvers, MA, USA; No. 84580T; 1:1000). HRP-conjugated goat anti-rabbit IgG (Cell Signaling Technology, Danvers, MA, USA; No. 7074P2; 1:1000) was used for signal detection, further performed with chemiluminescent HRP substrate (Merck KGaA, Darmstadt, Germany; Luminata Crescendo; No. WBLUR0500) and membrane imaging MicroChemii system (DNR Bio-Imaging System Ltd., Neve Yamin, Israel).

### 2.9. Cell Transfection and Transduction

Transfections were performed using Lipofectamine™ RNAiMAX (Thermo Fisher Scientific, Waltham, MA, USA; No. 13778100). Cells were seeded into 6-well plates at a density of 1 × 10^5^ cells per well 24 h before transfection. Immediately prior to transfection, cells were washed twice with PBS containing Mg^2+^ and Ca^2+^, and the medium was replaced with OptiMEM (Gibco, Grand Island, NY, USA; No. 31985062). Lipofectamine™ RNAiMAX/MISSION^®^ esiRNA (Invitrogen, Grand Island, NY, USA/Sigma-Aldrich, St. Louis, MO, USA) transfection complexes were prepared by mixing reagents immediately before use to yield a final esiRNA concentration of 25 pmol per well. EsiRNA stocks (dissolved in TE buffer; A&A Biotechnology, part of kit No. 021-50) were quantified using a NanoDrop spectrophotometer (Thermo Fisher Scientific, Waltham, MA, USA) prior to complex formation. Cells were incubated with the Lipofectamine/esiRNA complexes for 16–18 h, after which the medium was replaced with standard culture medium and cells were maintained for an additional 48 h. The following MISSION^®^ esiRNAs were used in the experiments: targeting MFN1 (No. EHU113211; Sigma-Aldrich, St. Louis, MO, USA), targeting MFN2 (No. EHU044111; Sigma-Aldrich), targeting ABCB1 (No. EHU131661; Sigma-Aldrich, St. Louis, MO, USA), targeting ABCC1 (No. EHU076261; Sigma-Aldrich, St. Louis, MO, USA), and targeting ABCG2 (No. EHU012921; Sigma-Aldrich, St. Louis, MO, USA). Quantitative fluorescence analysis was performed as described in Section 2.5.

For visualization of mitochondrial transfer, T98G cells were seeded under standard culture conditions onto Ibidi µ-dishes (35 mm) at a density of 5 × 10^4^ cells per dish 24 h prior to the experiment. CellLight™ Mitochondria-GFP, BacMam 2.0 (Thermo Fisher Scientific, Waltham, MA, USA; No. C10600) was added to the growth medium (10 µL per dish). After a 16 h incubation, cultures were washed twice with PBS containing Mg^2+^ and Ca^2+^ and exposed to DOX. At designated time points, the medium was replaced with FluoroBrite™ DMEM supplemented with 10% FBS and 1% GlutaMAX (Gibco^®^, Grand Island, NY, USA), and samples were imaged to visualize intercellular mitochondrial transfer using a Leica DMI6000B inverted microscope (Leica Microsystems GmbH, Wetzlar, Germany) equipped with a time-lapse module (37 °C, 5% CO_2_).

### 2.10. Metabolic Activity Measurements

The metabolic profile of cells was assessed using a Seahorse XFp analyser (Agilent, Santa Clara, CA, USA) following the manufacturer’s protocols. Twenty-four hours prior to measurement, cells cultured under the selected experimental conditions were seeded into dedicated assay plates at a density of 1.5 × 10^4^ cells/well. Immediately before the assay, standard culture medium was replaced with Seahorse XF DMEM (Agilent, Santa Clara, CA, USA) supplemented with 10 mM glucose, 1 mM pyruvate, 2 mM glutamine and 5 mM HEPES (pH 7.4), and cells were incubated for 45 min at 37 °C. Bioenergetic profiling was performed using the Mito Stress Kit (Agilent, Santa Clara, CA, USA; No. 103010-100), with final reagent concentrations applied in the wells as follows: 1.5 µM oligomycin A, 1 µM FCCP and 0.5 µM rotenone/antimycin A [33]. Oxygen consumption rate (OCR) traces were used for quantitative estimation of parameters describing cellular metabolic activity. Data were processed in Wave software (Agilent, Santa Clara, CA, USA; v. 2.6.3.5), including the application of the software’s active worksheets containing predefined calculation algorithms for the desired parameters. Results were normalized to the cell number in each well; for this purpose, cells were fixed with 3.7% formaldehyde, stained with Hoechst 33342, imaged using the tile-scan module of a Leica DMI6000B fluorescence microscope (Leica Microsystems GmbH, Wetzlar, Germany), and nuclei counts were obtained by image analysis in ImageJ (v. 1.54f).

### 2.11. Calcein Efflux Assay

Cells were seeded onto 12-well culture plates (Corning^®^ Costar^®^, Corning, NY, USA) at a density of 2 × 10^4^ cells/well and, after 24 h, incubated with Calcein-AM (Invitrogen, Grand Island, NY, USA; No. C3099,1 µg/mL) for 30 min. The medium was then replaced with FluoroBrite^®^ DMEM (Gibco^®^, Grand Island, NY, USA) supplemented with 10% FBS and 1% GlutaMAX (Gibco^®^, Grand Island, NY, USA). Baseline (t_0_) intracellular calcein fluorescence and time-courses of its changes were recorded using a Leica DMI6000B fluorescence microscope (Leica Microsystems GmbH, Wetzlar, Germany) equipped with an Alexa488 filter set and a time-lapse module (interval = 30 min; total acquisition time = 120 min). Acquired images were analyzed in ImageJ (v. 1.54f), and the resulting numerical data were used to quantify the kinetics of calcein fluorescence intensity normalized to the initial time point (t_0_). Data are representative of 3 independent biological replicates.

### 2.12. Measurements of Intracellular Metabolites Content

Cells were plated on 100 mm dishes at a density of 1 × 10^6^ cells per dish, subjected to a pulse DOX exposure and further culture, and then washed twice with PBS (37 °C) to remove residual medium. All subsequent sample-preparation steps were carried out on dry ice to preserve metabolite integrity. Metabolites were extracted using 500 µL of cold methanol at 80% (*v*/*v* in H_2_O; VWR, Radnor, PA, USA; HPLC standard). The methanol solution was pre-cooled to −80 °C for 2 h prior to use. The chilled solvent was gently dispensed to cover the entire culture surface, plates were tightly sealed with parafilm to prevent evaporation, and incubated at −80 °C for 30 min. Cells were then scraped from the vessel surfaces on dry ice using a sterile spatula. The resulting lysate, including remaining cellular debris, was transferred into pre-cooled Eppendorf (safe lock) tubes. To maximize recovery, the dish surfaces were rinsed several times with the extraction solvent and the rinses were pooled with the primary lysate. Prepared lysates were stored at −80 °C until analysis. Samples were transported to the analytical facility on dry ice to avoid uncontrolled thawing.

Subsequently, samples were sonicated for 10 min on ice, vortexed and centrifuged (15 min, 16,600× *g*, 4 °C). The supernatant was lyophilized from a volume of 400 μL. Lyophilized extracts were kept at −80 °C until LC-MS/MS analysis. DNA content in the obtained supernatant was determined using a NanoDrop Lite spectrophotometer (Thermo Scientific, Waltham, MA, USA). Samples were reconstituted in 2000 μL of 50% (*v*/*v*) acetonitrile containing an internal standard mixture, and 3 μL was injected onto the chromatographic column. Analyses were performed by LC-MS/MS using a QTRAP 5500 mass spectrometer (SCIEX, Framingham, MA, USA) coupled to a UFLC Nexera chromatograph (Shimadzu, Kyoto, Japan). Samples were analyzed in multiple reaction monitoring mode (MRM) in both negative and positive ionization (ESI; Electrospray). Chromatographic separation was achieved on a Discovery^®^ HS F5 HPLC Column, 3 μm, 2.1 × 100 mm, (Supelco (Sigma-Aldrich/Merck), Bellefonte, PA, USA) using gradient elution at a flow rate of 0.3 mL·min^−1^. The gradient program for negative-ion mode was: 0–3 min, 0% B; 3–25 min, 0 → 90% B; 25–34 min, 90% B; 34–35 min, 90 → 0% B; 35–40 min, 0% B, with mobile phases A: H_2_O + 1 mM acetic acid and B: acetonitrile + 1 mM acetic acid. The gradient program for positive-ion mode was: 0–3 min, 0% B; 3–31 min, 0 → 90% B; 31–39 min, 90 → 95% B; 39–40 min, 95 → 0% B; 40–45 min, 0% B, with mobile phases A: H_2_O + 0.1% formic acid and B: acetonitrile + 0.1% formic acid. Data acquisition and peak integration were performed using analyst software (SCIEX, Framingham, MA, USA). Quantitative results were normalized to total DNA content (n = 3 independent biological replicates). The entire sample preparation, this methodological description and the measurements were performed as a service by the Metabolomics Analysis Laboratory, Jagiellonian Centre for Experimental Therapeutics (JCET).

To assess intracellular ATP levels, cells were plated in 96-well glass-bottom plates at a density of 5 × 10^3^ cells per well, then subjected to the pulse DOX treatment/regeneration protocol. ATP quantification was performed using the ATP Determination Kit (Invitrogen, Grand Island, NY, USA; No. A22066) and measured with an Infinite 200 Pro plate reader (Tecan Group Ltd., Männedorf, Switzerland), following the manufacturer’s instructions [33]. For intracellular NADH measurement, cells were seeded into Petri dishes at a density of 2 × 10^6^ cells per dish and similarly processed through the pulse DOX treatment/regeneration protocol. The NAD/NADH ratio was determined using a dedicated biochemical kit (Sigma-Aldrich, St. Louis, MO, USA; No. MAK037). Prior to absorbance readings, samples were deproteinized using 10 kDa molecular weight cut-off filters at 4 °C and 14,000× *g*. Absorbance was then measured at the assay-specific wavelength using a Multiskan FC microplate reader (ThermoFisher Scientific, Waltham, MA, USA). All results were normalized to cell count (seeded on separate dishes), as determined by a Z2 particle counter (Beckman Coulter, Brea, CA, USA).

### 2.13. Statistical Analysis

Statistical analyses were performed using analysis of variance (ANOVA) with permutation-based FDR control (<0.05) followed by Tukey’s post hoc test or Student’s *t*-test with Benjamini–Hochberg FDR set to 0.05 (LC-MS/MS proteomics) and the nonparametric Mann–Whitney U test, implemented in Origin 2020 (v. 9.7.0.188; OriginLab Corporation, Northampton, MA, USA) or GraphPad Prism 8.4.0 (GraphPad, San Diego, CA, USA). *p*-values > 0.05 were considered indicative of no statistical significance. Error bars in figures represent either ± SEM (standard error of the mean) or ± SD (standard deviation), as indicated in the figure legends. For clarity of presentation, and unless otherwise stated, statistical significance was assessed between the sample and the corresponding control.

## 3. Results

### 3.1. Hallmarks of Mitochondrial Mobilization in DOX-Induced PGCs

**Figure 1:**
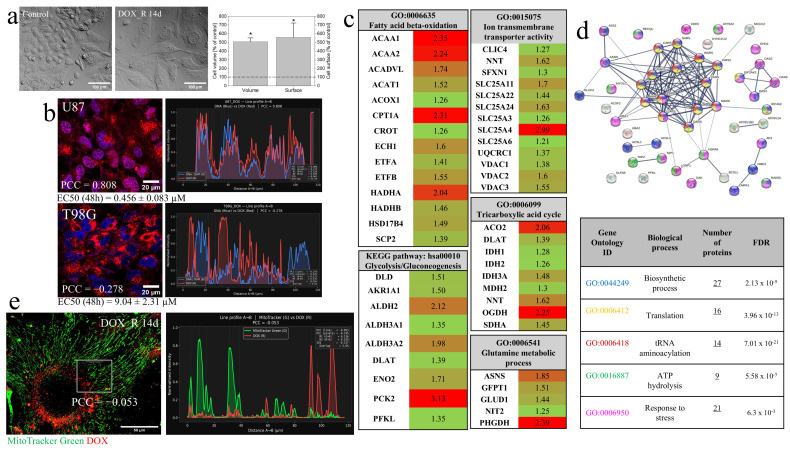
Metabolic mobilization of DOX-induced PGCs. (**a**) Morphology (left) and size (right) of pulse DOX-treated (1 µM; 48 h) T98G cells 14 days after DOX removal (averaged cell volume and surface projection area expressed as a % of control. Note the hypertrophy of DOX-treated T98G cells. Statistical significance was assessed by non-parametric Mann–Whitney test; * *p* < 0.05 versus control. Bars represent SD. Data are representative of ≥50 single cells in three independent biological replicates. Scale bars—100 µm. (**b**) Reverse correlation between nuclear DOX localization and IC_50_ values estimated for U87 and T98G cells. PCC values represent the degree of DOX- and DNA-specific fluorescence colocalization (Hoechst 33258; representative for >50 cells in 3 independent replicates). Scale bars—20 µm. (**c**) Heatmaps of metabolic proteins significantly upregulated in DOX_R 14d cells as determined by LC–MS/MS proteomics and classified according to Gene Ontology (GO) and KEGG pathway annotations. Heatmap colors reflect fold-increase versus control (red = highest; green = lowest). (**d**) Interactome of ATP-dependent proteins significantly increased in DOX_R 14d cells (Fold change ≥ 1.2). Interaction networks were generated using the STRING database; high-confidence interaction score threshold (0.700). The table below the network assigns individual proteins to biological processes according to GO. Statistical significance was assessed by Student’s *t*-test with Benjamini–Hochberg FDR set to 0.05 (*p* < 0.05 vs. control). Data are representative of three independent biological replicates. (**e**) Extra-mitochondrial localization of DOX in T98G cells on the 14th day after DOX removal estimated by confocal microscopy. An exemplary PCC value illustrates a low degree of DOX- and MitoTracker Green-specific fluorescence co-localization. Data are representative of ≥50 single cells in three independent biological replicates. Scale bar—50 µm. Note the concomitant extra-nuclear and extra-mitochondrial DOX sequestration, mobilization of mitochondrial catabolism, and mobilization of cell anabolic machinery.

Pulse treatment of T98G cells with doxorubicin (DOX; 1 µM, 48 h) induces their biphasic adaptive response. In the first (“remission”) phase, a transient DOX-induced epithelial–mesenchymal transition (EMT) is followed by extensive cell death and the concomitant emergence of polymorphonuclear “giant” cells (PGCs) among the surviving cells. In the second (“relapse”) phase, PGCs support the propagation of newly formed clusters of proliferative non-PGCs [33]. PGCs display relatively high volumes, surface projection areas (>5000 um^2^); therefore, the emergence of PGCs in DOX-treated T98G populations is manifested by increased averaged values of both parameters (Figure 1a; [33]). The longevity of PGCs results from their efficient management of DOX-induced stress, manifested by extranuclear DOX compartmentation (Figure 1b). It was considerably more efficient than in DOX-sensitive U87 cells. Concomitantly, we observed the mobilization of the catabolic (ATP-generating) machinery, including glutamine/glutamate transport, upregulation of glycolytic/glucogenic and auxiliary systems, such as β-oxidation, the tricarboxylic acid cycle, the glutaminolytic axis (Figure S1a–d), and numerous mitochondrial ion transmembrane transporters incl. voltage-dependent anion channels (VDACs; Figure 1c and Figure S1e,f). It was paralleled by the up-regulation of a broad cluster of proteins constituting anabolic (ATP-consuming) systems (Figure 1d). Together with the apparent DOX sequestration in extra-mitochondrial compartments (cytoplasmic vesicles; Figure 1e; cf. Figure S2), these data suggested the vital role of mitochondrial welfare in the intracellular DOX compartmentation and DOX-resistance of T98G cells.

### 3.2. Mitochondrial Adaptation to DOX-Induced Stress

**Figure 2:**
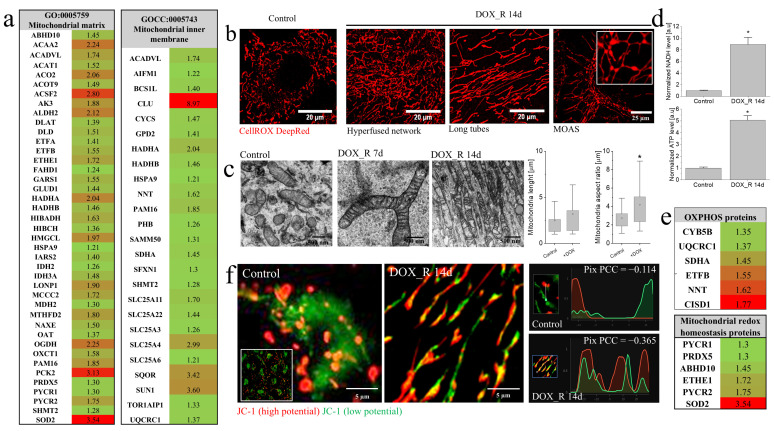
Mitochondrial dynamics in pulse DOX-treated T98G cells. (**a**,**e**) Heatmaps of mitochondrial matrix/inner membrane (**a**), oxidative phosphorylation and mitochondrial stress proteins (**e**) significantly upregulated in DOX_R 14d cells and determined by LC–MS/MS proteomics and classified according to Gene Ontology (GO) and KEGG pathway annotations. Heatmap colors reflect fold-increase versus control (red = highest; green = lowest). Statistical significance was assessed by ANOVA with permutation FDR < 0.05 and Tukey post hoc (*p* < 0.05 vs. control). Data are representative of three independent biological replicates. (**b**) Structural variants of mitochondria (HNs, LTs and mitochondria-on-a-string; MOAS) visualized in control and DOX_R 14d cells by CellROX DeepRed staining (upper panel) and the quantification of mitochondrial length and aspect ratio estimated with custom Python pipeline based on the Frangi tubeness filter (lower panel). Scale bars—20 and 25 µm (**c**) Ultrastructure of mitochondria in DOX_R 14d c ells visualized via transmission electron microscopy (TEM). Scale bars—500 nm (**d**) NADH and ATP levels in DOX_R 14d cells quantified with ATP content assay. (**f**) Mosaic distribution of mitochondrial membrane potential along tubular networks visualized with JC-1 staining and a custom Python pipeline. Pix PCC values indicate co-localization of green/red fluorescence within individual pixels. Scale bars—5 µm. Statistical significance of the differences in (**b**,**d**) was assessed by non-parametric Mann–Whitney test; * *p* < 0.05 vs. control. Bars represent SD. Data are representative of three independent biological replicates and ≥50 single cells. Note the signs of mitochondrial adaptation in PGCs under DOX-induced stress.

The apparent significance of mitochondria for the welfare of DOX-treated T98G cells prompted us to focus on the processes involved in mitochondrial adaptation to metabolic and chemotherapeutic stress induced by a pulse DOX treatment. Proteomic analyses identified a cluster of upregulated “mitochondrial matrix” and/or “mitochondrial inner membrane” proteins on the 14th day following pulse DOX treatment of T98G cells (Figure 2a). Mobilization of their mitochondria was also manifested by the formation of elongated, tubular mitochondria (Figure 2b; for visualization of their ultrastructure, see Figure 2c), the dynamics of their networks (Figure 2b and Figure S3a) and the “mitochondria-on-a-string” (MOAS; Figure 2b and Figure S3b). Concomitantly, we observed ATP accumulation (Figure 2d; [33]), up-regulation of “OXPHOS” and “mitochondrial stress” proteins (Figure 2e), the appearance of “mosaic” mitochondrial networks, characterized by a heterogeneous distribution of membrane potentials (Figure 2f), and extensive intercellular trafficking of mitochondria mediated by tunneling nanotubes (TNT; Figure S3c). Collectively, these data show the adaptive nature of the mitochondrial dynamics and the efficiency of the mechanisms governing PGC mitochondrial welfare under DOX-induced stress.

### 3.3. Mitofusin(MFN)2 Mobilization in T98G Cells Under DOX-Induced Stress

**Figure 3:**
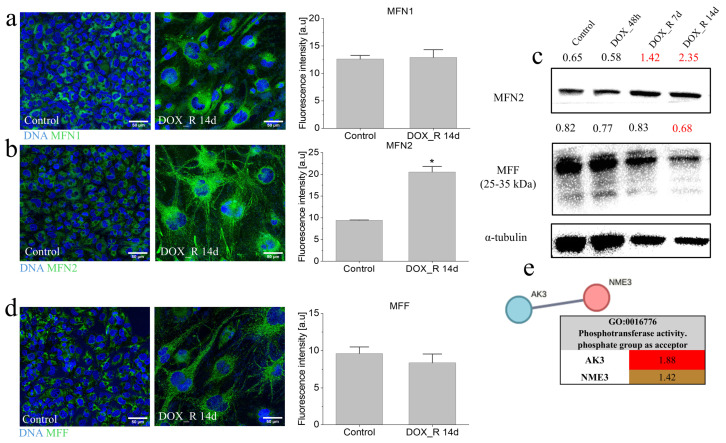
Mitofusin 2 up-regulation in pulse DOX-treated T98G cells. (**a**,**b**,**d**) MFN1 (**a**), MFN2 (**b**) and MFF (**d**) levels in pulse DOX-treated T98G cells (1 µM; 48 h) estimated with quantitative fluorimetry at the 14th day after DOX removal. Data are representative of 3 independent biological replicates. Bars represent SD. Statistical significance of the differences was assessed by non-parametric Mann–Whitney U test; * *p* < 0.05 vs. control (**a**,**b**,**d**). Scale bars—50 µm. (**c**) MFN2 and MFF levels visualized in pulse DOX-treated T98G cells with (1 µM; 48 h) estimated with immunoblotting. Numerical values represent results of densitometric analyses, normalized against housekeeping protein levels (α-tubulin) and compared to control. (**e**) Interactome of ATP/GTP conversion proteins significantly up-regulated in DOX_R 14d cells as estimated by LC–MS/MS proteomics (Fold change ≥ 1.2). Interaction networks were generated using the STRING database (high-confidence interaction score threshold—0.700). The table below assigns individual proteins to biological processes according to GO. Statistical significance was assessed by Student’s *t*-test with Benjamini–Hochberg FDR set to 0.05 (*p* < 0.05 vs. control) from experimental triplicates (n = 3). Note the adaptive up-regulation of MFN2 in PGCs.

In a further search of the potential regulators of the mitochondrial dynamics in T98G cells under DOX-induced stress, we pinpointed mitofusins (MFNs), GTPase-activity proteins known to mediate mitochondrial fusion in stress conditions [47,48]. Immunofluorescence analyses confirmed the presence of both mitofusin 1 (MFN1; Figure 3a) and mitofusin 2 (MFN2; Figure 3b) in DOX-induced PGCs (DOX_R 14d). While MFN1 levels remained unchanged following a pulse DOX treatment of T98G cells, quantitative fluorimetric analysis of MFN2 revealed its significant induction under these conditions. These data were supported by qualitative immunoblot studies (Figure 3c). Concomitantly, we observed the downregulation of mitochondrial fission factor (MFF) in PGCs (Figure 3c,d), whereas no changes in OPA1 levels were observed (Figure S4).

These findings suggest that the balance between MFN2 and MFF activity governs the dynamics of adaptive mitochondrial fusion and networking in T98G cells undergoing a pulse DOX treatment. These shifts were accompanied by an upregulation of nucleoside diphosphate kinase 3 (NME3) and adenylate kinase 3 (AK3) following pulse DOX treatment of T98G cells (Figure 3e). Given the role of NME3 and AK3 in ATP–GTP homeostasis, MFN2 induction appears to participate in reinforcing mitochondrial welfare and metabolic adaptation of T98G cells to DOX-induced stress.

### 3.4. Consequences of MFN2 Down-Regulation in the Absence of DOX

**Figure 4:**
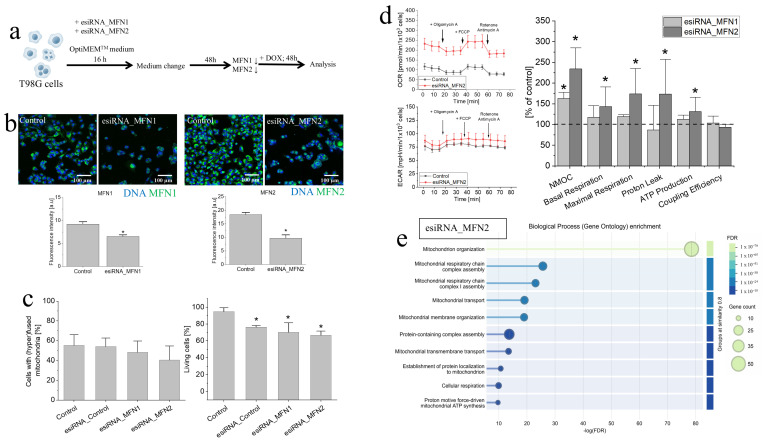
The effect of MFN1/2 down-regulation on mitochondrial homeostasis in T98G cells. (**a**) Experimental approach to the estimation of the consequences of MFN1/2 silencing in T98G cells. Cells were treated with the relevant esiRNAs for 16 h and cultivated for 48 h before endpoint analyses. (**b**) MFN1 or MFN2 levels following the treatment of T98G cells with esiRNA estimated by quantitative fluorimetry. Scale bars—100 µm. (**c**,**d**) Effect of MFN1 or MFN2 down-regulation in T98G cells on the morphology of their mitochondria (**c**) (**left**), the fraction of viable T98G cells (Trypan blue assay); (**c**) (**right**), and metabolic profile (**d**). Statistical significance was assessed by non-parametric Mann–Whitney test; * *p* < 0.05 vs. control. Data are representative of 3 independent biological replicates and/or ≥50 single cells. (**e**) Mitochondrial protein levels in T98G cells undergone MFN2 down-regulation quantified by LC–MS/MS; proteins with fold change >1.2 were classified into functional clusters in the STRING database according to Gene Ontology. Statistical significance was assessed by Student’s *t*-test with Benjamini–Hochberg FDR set to 0.05 (*p* < 0.05 vs. control). Data are representative of 3 independent biological replicates. Note a slight deterioration of T98G welfare accompanied by respiratory mobilization of T98G cells following MFN2 down-regulation.

To further address the involvement of mitofusins in the regulation of mitochondrial T98G homeostasis, we transiently down-regulated MFN1 and MFN2 in T98G cells by esiRNA (Figure 4a). In short-term analyses, MFN1- and MFN2-targeted esiRNA treatment considerably down-regulated both proteins in T98G cells, as illustrated by fluorescence microscopy-assisted quantitative fluorimetric studies (Figure 4b). However, MFN2-targeted esiRNA only slightly decreased the number of T98G cells displaying hyperfused mitochondrial networks, whereas MFN1 down-regulation exerted no detectable effect on the efficiency of this process (Figure 4c). Concomitantly, a slightly decreased fraction of viable cells was observed in T98G populations undergoing MFN1/MFN2 down-regulation. Seahorse analyses of the metabolic profile revealed a general lack of the effect of MFN1 depletion on the respiration of T98G cells (Figure 4d, cf. Figure S5). Surprisingly, we observed the signs of OXPHOS mobilization (Figure 4d, cf. Figure S6), increased proton leak and a robust proteomic reprogramming of mitochondrial networks following MFN2 down-regulation (Figure 4e; cf. Figures S7 and S8). This is illustrated by the mobilization of mitochondrial stress management, membrane organization and respiratory systems. In particular, we observed up-regulated MFN1 levels (Figure 4d; cf. Figure S8, red circle). Collectively, these data show the involvement of MFN2 in the regulation of mitochondrial T98G homeostasis but also indicate the redundancy of the systems that determine this process.

### 3.5. MFN2 Down-Regulation Interferes with the Early T98G Adaptation to DOX-Induced Stress

**Figure 5:**
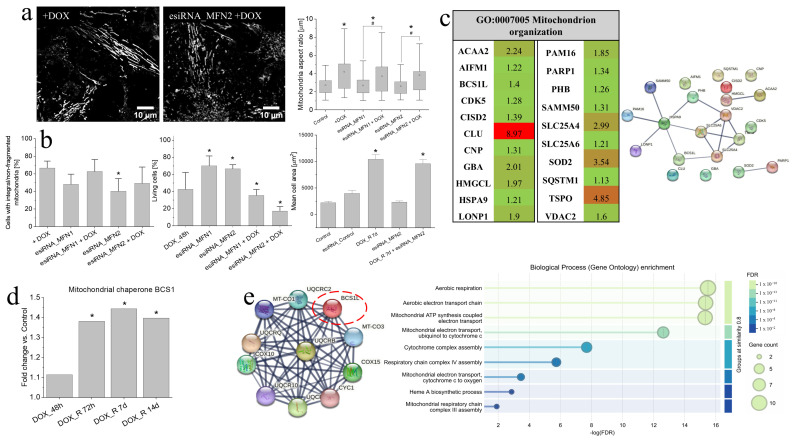
MFN2 involvement in the mitochondrial homeostasis of T98G cells following their pulse DOX treatment. (**a**) Effect of MFN1 or MFN2 down-regulation on the morphology of their mitochondria following pulse DOX (48 h) treatment (measured as a fraction of T98G cells that display the presence of integral/non-fragmented mitochondria; Python pipeline-assisted analysis; cf. Figure S9). Scale bars—10 µm (**b**) Effect of MFN1 or MFN2 down-regulation on mitochondrial organization in T98G cells (**left**), the fraction of viable T98G cells following a pulse DOX treatment (estimated with Trypan blue assay; middle) (and surface area; **right**). Statistical significance was assessed by non-parametric Mann–Whitney test; * *p* < 0.05 vs. control and # *p* < 0.05 vs. DOX-treated variant. Data are representative of ≥50 single cells in 3 independent biological replicates. (**c**) Heatmap of upregulated “mitochondrion organization” proteins estimated with LC-MS/MS proteomics in DOX_R 14d cells (red = highest increase within each group; green = lowest increase; fold change > 1.2). (**d**) BCS1 (BCS1L) levels in T98G cells following their pulse DOX treatment at specific time points, estimated with LC-MS/MS. (**e**) An enriched interactome between BCS1L (red dashed circle) and its closest physical interactors generated using the STRING database and a high-confidence interaction score threshold (0.700). The diagram on the right represents Gene Ontology-based functional enrichment analysis of the abovementioned proteins, which identified a coherent group of biological processes collectively associated with mitochondrial bioenergetics regulation. Statistical significance was assessed by ANOVA with permutation FDR < 0.05 and Tukey post hoc (*p* < 0.05 vs. control). Data are representative of 3 independent biological replicates. Note the presence of tubular mitochondria in minute T98G cell sub-populations that survived a combined MFN2 down-regulation/DOX treatment, accompanied by the up-regulation of other regulators of mitochondrial organization.

Up-regulation of MFN2 in DOX-induced PGCs (cf. Figure 3), accompanied by increased OXPHOS intensity following MFN2 down-regulation and its negligible effect on T98G welfare in the absence of DOX (cf. Figure 4) prompted us to focus on MFN2 involvement in the adaptation of T98G cells to pulse DOX treatment. MFN1- and MFN2-targeted esiRNA treatment failed to induce a global dismantling of the mitochondrial tubes and hyperfused mitochondrial networks following a pulse DOX treatment. This is illustrated by the averaged values of mitochondrial aspect ratio (Figure 5a; cf. Figure S9) and % of cells displaying hyperfused mitochondria following MFN1/2 down-regulation, which remained similar to control (ca. 40–50%; Figure 5b; cf. Figure 4c). In turn, pulse DOX treatment increased the T98G fraction displaying hyperfused mitochondria to >60% and concomitant MFN1 or MFN2 down-regulation exerted a negligible effect on the efficiency of DOX-induced mitochondrial fusion. Only a slightly impaired DOX-induced, mitochondrial fusion-related response was observed following the knockdown of MFN2 in T98G cells. Notably, the numbers of DOX-treated T98G cells with hyperfused mitochondria remained higher than in the absence of DOX. In parallel, we observed reduced numbers of T98G cells, which were capable of surviving a combined MFN2 down-regulation/DOX-induced stress (Figure 5b). However, these cells retained high surface areas characteristic of PGCs. These data indicate the involvement of compensatory “chaperon” systems in sustaining mitochondrial homeostasis of DOX-treated T98G cells. Actually, we observed a cluster of up-regulated “mitochondrial organization proteins” in T98G cells following a pulse DOX treatment (Figure 5c). In particular, T98G cells reacted to DOX-induced stress with the up-regulation of BCS1L (Figure 5d), and the cluster of proteins involved in the preservation of the respiratory chain integrity (Figure 5e). In conjunction with the mobilization of mitochondrial bioenergetics (cf. Figure 1 and Figure 2 [33]) and apparent DOX exclusion from mitochondrial networks in PGCs (cf. Figure 1), these data indicate the redundancy of mitochondrial protection systems in T98G cells.

### 3.6. Activity of DOX Relocation/Retention Axis Following MFN2 Down-Regulation

Our data indicate that metabolic reprogramming secures the energy resources for the intracellular DOX relocation/retention axis in T98G cells (Figure 1b,e; [33]). The activity of this axis in T98G cells is manifested by the sustained presence of DOX in T98G cells following a pulse DOX treatment [33]. The apparent redundancy of the regulators of mitochondrial dynamics prompted us to estimate the links of MFN2 function with the DOX relocation/retention axis in T98G cells.

**Figure 6:**
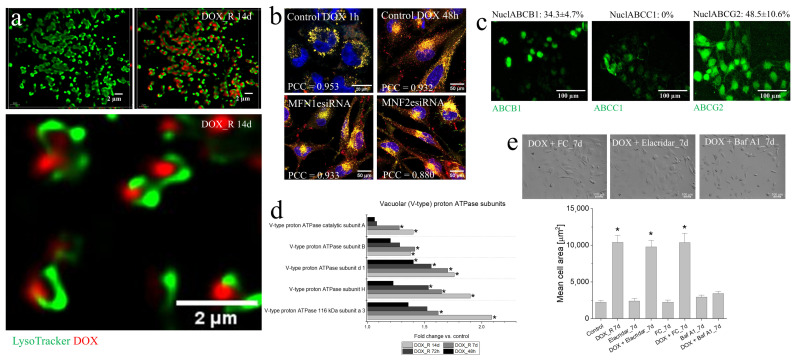
MFN2 and the activity of the drug-relocation/retention system in DOX-resistant cells. (**a**) Lysosomal DOX retention (red) in pulse DOX-treated (1 µM; 48 h) T98G cells visualized by LysoTracker and confocal microscopy on the 14th day after DOX removal (representative images for 3 independent biological replicates). (**b**) Effect of MFN2 silencing on lysosomal DOX retention in T98G cells. (**c**) Immunolocalization of selected ABC transporters in T98G cells; accompanying values show the percentage of cells exhibiting nuclear localization for each ABC protein. (**d**) V-type proton ATPase levels in T98G cells following their pulse DOX treatment estimated with LS-MS. Statistical significance was assessed by ANOVA with permutation FDR < 0.05 and Tukey post hoc (*p* < 0.05 vs. control) from experimental triplicates (N = 3). (**e**) Effect of metabolic inhibitors, MFN1/2 silencing and ABC/V-type proton ATPase inhibitors on the formation of PGCs in T98G populations under DOX-induced stress. Scale bars = 2 (**a**), 20 and 50 (**b**) and 100 µm (**c**,**e**). Statistical significance was calculated with non-parametric Mann–Whitney U test, * *p* < 0.05 vs. control (**e**). All data are representative of 3 independent biological replicates. Error bars represent SD values. Note the lysosomal accumulation of DOX in PGCs and impairment of the PGC program following the Baf A1 administration.

The co-localization of DOX with the intracellular Lysotracker^+^ vesicles (Figure 6a; cf. Figure S10) demonstrated that the long-term DOX retention in T98G cells (14 days after the pulse treatment) resulted from the accumulation and sequestration of this drug in lysosomes. However, MFN2-deficient T98G cells retained lysosomal DOX accumulation (Figure 6b; cf. Figures S11 and S12), which confirms the redundancy of the systems linking cell metabolism and the DOX retention system in T98G cells (i.e., a “resistance triad”). Further analyses showed nuclear localization of ABCB1 and ABCG2 in T98G cells (Figure 6c) and their redundancy (illustrated by negligible effects of ABCB1 inhibition on calcein efflux and T98G DOX sensitivity; cf. Figure S13). In turn, proteomic studies of DOX-induced PGCs revealed the up-regulation of a cluster of proton pumps (V-ATPases; Figure 6d). These observations indicate that ABCB1 and ABCG2 determine extra-nuclear DOX transport in T98G cells, whereas V-ATPases sequestrate DOX by acidifying the lysosomal lumens, creating proton gradients, and maintaining lysosomal membrane potential and DOX protonation [58,59]. The DOX-induced PGC program is manifested by T98G hypertrophy (cf. Figure 1a and [33]). Therefore, the analyses of the effect of MFN2 down-regulation and chemical inhibition of ABCB1, ABCG2 and V-ATPases (by Elacridar, Fumitremorgin C and Bafilomycin A1, respectively) on the projection areas of T98G cells enabled us to link their function with the PGC program. Similarly to MFN2 down-regulation, ABCB1 and ABCG2 inhibitors did not interfere with the PGC program (Figure 6e and Figure S14). In turn, its progression was apparently impaired by V-ATPases inhibition. These observations demonstrate the significance of lysosomal V-ATPases for lysosomal DOX sequestration in T98G cells and the redundancy of the T98G “resistance triad” which preserves their homeostasis under chemotherapeutic stress.

## 4. Discussion

Poly(morpho)nuclear “giant” cells (PGCs) were originally regarded as “decadent products” of genome degradation in intoxicating conditions. However, their protective and generative potential has more recently been implicated in the adaptation of tumor ecosystems to microenvironmental stress [60,61,62]. Being characterized by multiplied genome, hypertrophy and the mobilization of self-defense systems, PGCs can serve as the reservoir of genetic diversity and chemoresistance of tumor ecosystems. Our previous studies demonstrated metabolic mobilization and resistance of PGCs to chemotherapeutic burden during the adaptation of glioblastoma multiforme (GBM) T98G populations to DOX-induced stress [33]. These findings stay in contrast to the high DOX-sensitivity of other cellular GBM models (i.e., U87) and identify T98G cells as a unique model, suitable for the analyses of the processes underlying the links between mitochondrial dynamics, metabolic plasticity and the activity of drug-resistance systems in GBM. Mitochondrial fusion (networking) that we have observed in T98G PGCs under DOX-induced stress prompted us to focus on its links with the adaptation of T98G cells to DOX-induced stress. Here, (i) we report the significance of a “triad” of mitochondrial networking/activation, ABC transporter-dependent perinuclear DOX redistribution and V-type channel-mediated lysosomal DOX compartmentalization as a system that determines DOX resistance of T98G cells. We also show the data that (ii) indicate MFN2 contribution to the mitochondrial dynamics and T98G adaptation to increased metabolic and chemotherapeutic burden. However, (iii) the redundancy of mitochondrial chaperons and ABC transporters in T98G cells indicates the limited potential of MFN2 and other “resistance triad” elements as therapeutic targets. 

Metabolic reprogramming of PGCs, the activation of the drug relocation axis and the mobilization of intracellular drug-retention systems in T98G PGCs constitute a functional “resistance triad” that can further initiate the recovery of GBM populations from chemotherapeutic stress. Metabolic flexibility of GBM cells has long been regarded as the determinant of GBM drug-resistance under fluctuating oxygen and nutrient conditions typical of the tumor microenvironment [63,64]. Our current findings show the mobilization of the mitochondrial proteome and the participation of glycolytic and glutaminolytic pathways in the metabolic reprogramming of T98G cells. These auxiliary systems cooperate with mobilized β-oxidation, the Krebs cycle and oxidative phosphorylation (OXPHOS) to ensure energy supply for the agitated drug-resistance systems [65,66,67]. Concurrent mitochondrial rearrangements that we observed in DOX-induced T98G PGCs (i.e., mitochondrial fusion [51], cristae remodeling [68,69], intercellular transfer of mitochondria via TNTs [70,71], and the formation of mitochondrial networks [72,73]) further enhance adaptive capacities of mitochondria (stress-induced mitochondrial hyperfusion (SIMH; [74]). During this process, mitochondria fuse into long, interconnected networks that display more efficient uptake of energetic substrates (e.g glutamine), intensified conversion towards reductive and energetic carriers (NADH/NADPH/ATP) [75,76] and the mobilization of ROS scavenging systems [33]. Whereas further studies are necessary to determine the involvement of mitochondrial rearrangements in other GBM models, this process seems important for the metabolic plasticity of T98G PGCs as the 1^st^ element of the “resistance triad”.

In general, stress-induced metabolic reprogramming secures a stable energy supply for drug-resistance systems of cancer cells. However, drug-resistance of T98G PGCs is apparently related to the function of drug relocation and lysosomal retention systems, which depend on the activity of ABC transporters and lysosomal V-dependent proton pumps, respectively. ABC (ATP-binding cassette) transporters relocate DOX from peri-nuclear T98G compartments into the proximity of PGC lysosomes, whereas vacuolar (V)-proton pump ATPase(s) participate in the DOX protonation and trapping in acidic lysosomal compartments [77,78]. In our hands, their considerable up-regulation in DOX-treated T98G cells correlated with DOX accumulation in lysosomal PGC compartments and DOX absence in cell nuclei, mitochondria and the endoplasmic reticulum. This observation stays in contrast to U87 cells, where nuclear DOX accumulation was observed. Mitochondrial rearrangements can protect the welfare of mitochondria under increased metabolic and cytotoxic burden, maintaining bioenergetic homeostasis of PGCs. Thus, they (i) secure the energy supply for ABC transporters and lysosomal (V)-proton pump ATPases [58,77,78], sustaining the T98G ability to (ii) relocate and (iii) compartmentalize DOX. These systems cooperatively reduce the intracellular bioavailability of DOX, impairing its cytotoxic effects, and constituting the second and third elements of the T98G “resistance triad”, respectively. Its significance for T98G adaptation to DOX-induced stress is illustrated by the deteriorated welfare of PGCs following the inhibition of OXPHOS [33,79]. The inhibitors of glutaminolysis [80], ABC transporters and V-pumps [81] interfered with drug resistance in other cellular models. Cancer cells can resist therapy through drug exclusion and drug retention. During drug exclusion, cells reduce intracellular drug levels by exporting compounds via ABC transporters. In contrast, drug retention allows drugs to accumulate but minimizes their toxicity through intracellular sequestration. The general significance of the PGC “resistance triad” for the adaptation of GBM cell lineages requires further experimental verification. However, our data indicate that it can minimize DOX bioavailability in critical cytoplasmic compartments and in the extracellular environment of T98G PGCs, which represent an in vitro model of drug-tolerant persister cells [59,81,82].

Our notion of the crucial role of mitochondrial fusion as the core of the T98G “resistance triad” was further supported by MFN2 regulation in T98G cells following a pulse DOX treatment. It plays a key role in mitochondrial dynamics, specifically in the process of mitochondrial fusion and aberrant ER-mitochondrial tethering in cancer cells, which enhances regenerative mitochondrial potential and cancer drug-resistance [46,47,48]. This process is generally associated with enhanced OXPHOS [83,84]. However, the fragmentation of mitochondria can also sustain/elevate OXPHOS [85], whereas the fusion events occasionally suppress OXPHOS to promote metabolic quiescence under stress [86,87,88,89,90]. In our hands, mitochondrial fusion followed the initial mitochondrial “burst” and was accompanied by gradual decrease in mitochondrial activity in T98G PGCs between the second and fourteenth day after DOX removal [33]. The upward trend of MFN2 levels, parallel MFF down-regulation, a non-uniform mitochondrial distribution of MFN2/MFF, the accumulation of ATP and the mobilization of the ATP-GTP management system during the progressive formation of mitochondrial networks in PGCs may indicate the contribution of MFN2 to the mitochondrial welfare in DOX-induced PGCs [33]. Respiratory mobilization and deterioration of T98G welfare following MFN2 down-regulation suggest that MFN2-dependent mitochondrial networking (and possibly also ER-tethering), can partly mitigate mitochondrial respiratory stress. Small fractions of T98G cells that survive a combined MFN2 down-regulation and DOX treatment indicate that even a subtle disturbance of the naturally redundant “resistance triad” may evoke its critical dysfunction in a majority of T98G cells. It can interrupt early stages of GBM adaptation to DOX-induced stress, delaying the GBM regeneration in the long-run. In turn, efficient mitochondrial networking and PGC morphology of this minute fraction of T98G cells that survived a combined DOX/MFN2esiRNA treatment indicate the redundancy of this system. More comprehensive studies, employing (i) T98G cells engineered to display a stable MFN2 knock-out and (ii) more detailed and complementary analyses of the time-course of MFN2 down-regulation following esiRNA treatment are necessary to verify this notion. However, even with the transient MFN2 deficiency model, we observed the up-regulation of the cluster of “mitochondrial organization and chaperon” proteins, incl. mitochondrial chaperone and respiratory chain assembly factor BCS1L. Given its pivotal role in complex III biogenesis, OXPHOS and in the maintenance of tubular mitochondrial morphology, BCS1L upregulation appears as a candidate for alternative mechanism of mitochondrial adaptation to metabolic and chemotherapeutic stress. Collectively, MFN2 may help to build up the adaptive capacities of the T98G mitochondrion, enhancing its metabolic plasticity and securing an energy supply for the “resistance triad”. However, compensation of MFN2 function by other mechanisms can protect the mitochondrial welfare during early stages of T98G adaptation to DOX-induced chemotherapeutic stress and metabolic burden.

Adaptive plasticity of the T98G “resistance triad” is also enhanced by the redundancy of the intersections at the interface between the MFN2-dependent mitochondrial adaptive system and the DOX-relocation/retention axis. Namely, multi-level redundancy of the T98G “resistance triad” is manifested by negligible effects of the combined chemical ABCB1/G2 inhibition and pulse DOX treatment on the PGC program in T98G cells that had survived a combined ABC inhibition. Apparently, it helps integrate the metabolic reprogramming with the drug-relocation and drug-retention axis in intoxicated cells. Small PGC sub-populations showing the efficient “resistance triad” can drive GBM recovery following a pulse DOX treatment and act as the focal point of DOX-induced microevolution of “super-resistant” tumors, potentially limiting the potential of MFN2 and other “resistance triad” elements as targets for therapeutic strategy. A more prominent inhibition of the PGC program in T98G populations was manifested by reduced values of averaged T98G surface areas in the presence of DOX and Bafilomycin A1. It confirms the involvement of vacuolar-type H(+)-ATPases in DOX retention and might suggest the potential of these proteins as therapeutic targets. However, the plasticity of a “resistance triad” as a mechanistic entity encompassing mitochondrial adaptation and the drug-relocation/retention axis may still represent a major obstacle for the application of doxorubicin and, potentially, of other cytostatic drugs in the treatment of drug-resistant GBM variants.

## 5. Conclusions

Collectively, our observations show that metabolic reprogramming, the activation of drug-relocation and drug-retention facilitate the adaptation of T98G cells to DOX-induced stress. They add to previous reports on the links between metabolic plasticity and drug-resistance of GBM cells [6,33], which were based on the intuitive assumption claiming that ATP resources are necessary for the efficient action of drug-neutralization and efflux systems. Our approach reaches beyond this flat assumption because it is focused on “where”, “how” and “what for” such a network can be constituted. Accordingly, the mitochondrial adaptation to DOX-induced stress and the fine-tuning of mitochondrial metabolism secures ATP resources for the cooperative ABCB1, ABCC1 and ABCG2-dependent DOX-relocation and V-type H^+^ ATPase extranuclear DOX sequestration system in T98G cells. An open question remains whether this “resistance triad” can be considered as a target for novel combined/metronomic therapies. Apart from the problems concerning the drug application to GBM loci, the targeting of the “triad” may lead to the selective expansion of “super-resistant” lineages from the drug-resistant “elite” of heterogeneous GBM populations. Given (i) the heterogeneity of GBM, (ii) the contribution of therapeutic stress to GBM microevolution, and (iii) the versatility of drug-resistance strategies employed by discrete GBM cell lineages, our study conceivably describes only one of the scenarios responsible for malignant GBM remissions after chemotherapy. The significance of such “resistance triads” needs to be validated in other cellular systems, using the biopsy-derived GBM lines and/or the in vivo animal models. Finally, MFN2 may contribute to the mitochondrial adaptation to increased metabolic demand; however, ATP resources required for DOX compartmentalization can be secured by complementary adaptative mechanisms. Similarly, the redundancy of ABC transporters may add to the plasticity of the T98G “resistance triad”, further limiting its potential as a therapeutic target. Therefore, its significance should be verified using permanent MFN2/ABC knock-out models and more rigorous analyses of MFN2/ABC dynamics in intoxicated cells, using the techniques complementary to fluorimetry. However, the identification of the networks responsible for the adaptation of GBM cells to metabolic and chemotherapeutic stress and their malignant microevolution remains a promising route to understanding the background of GBM recurrences after chemotherapy.

## Data Availability

The data presented in this study are openly available in RODBUK at https://doi.org/10.57903/UJ/K33I4L. Python codes are available from the authors on reasonable request.

## Supplementary Materials

The following supporting information can be downloaded at https://www.mdpi.com/article/10.3390/cells15100899/s1, Figure S1: Mobilization of glutaminolytic axis and VDACs in T98G cells under DOX-induced stress; Figure S2: Experimental approach for the analysis of DOX (red) and MitoTracker Green signals in T98G_DOX14d cells; Figure S3: Mitochondrial dynamics in pulse DOX-treated PGCs; Figure S4: Effect of pulse DOX treatment (1 µM; 48 h) on OPA1 levels on the 14th day after DOX removal; Figure S5: The effect of MFN1 down-regulation by MFN1esiRNA on the parameters of mitochondrial respiration estimated with Seahorse XFp (MitoStress assay) 48 h after the transfection; Figure S6: The effect of MFN2 down-regulation by MFN1esiRNA on the parameters of mitochondrial respiration estimated with Seahorse XFp (MitoStress assay) 48 h after the transfection; Figure S7: Interactomes of (i), cellular response to stress” (ii), mitochondrial membrane organization” and (iii), oxidative phosphorylation” proteins significantly up-regulated in T98G cells undergoing MFN2 down-regulation and quantified by LC–MS/MS; Figure S8: Interactome of proteins involved in “mitochondria organization”, which were significantly up-regulated in T98G cells undergoing MFN2 down-regulation and quantified by LC–MS/MS; Figure S9: Analytic approach towards the estimation of the effect of MFN1 or MFN2 down-regulation on the morphology of T98G mitochondria following pulse DOX treatment; Figure S10: Dynamics of DOX sequestration in T98G cells; Figure S11: Experimental approach for the analysis of the co-localization of DOX (red) and LysoTracker signals in T98G cells undergoing MFN1 or MFN2 silencing; Figure S12: Experimental approach for the analysis of the co-localization of DOX (red) and LysoTracker signals in T98G cells (control; upper row) undergoing MFN1 (middle) or MFN2 silencing (lower row); Figure S13: Redundancy of the DOX relocation system in T98G cells; Figure S14: Effect of chemical ABCB1 and V-type proton ATPase inhibitors on the morphology (PGC formation) in T98G populations under DOX-induced stress.

## Abbreviations

The following abbreviations are used in this manuscript:

ABC	ATP-binding cassette
ABCB1	ATP-binding cassette sub-family B member 1
ABCC1	ATP-binding cassette sub-family C member 1
ABCG2	ATP-binding cassette sub-family G member 2
AK3	Adenylate kinase 3
ATCC	American Type Culture Collection
ATP	Adenosine triphosphate
BCS1L	Mitochondrial chaperone/assembly factor
BFA	Bafilomycin A/bafilomycin A1
BPTES	Glutaminase inhibitor
CMPK1	CMP kinase 1
Cx43	Connexin43
DMEM	Dulbecco’s Modified Eagle Medium
DMSO	Dimethyl sulfoxide
DOX	Doxorubicin
EMT	Epithelial–mesenchymal transition
ESI	Electrospray ionization
FASP	Filter aided sample preparation
FBS	Fetal bovine serum
FCCP	Carbonyl cyanide p trifluoromethoxyphenylhydrazone
GBM	Glioblastoma multiforme
GMT	Glial-mesenchymal transition
GO	Gene Ontology
GSK-3β	Glycogen synthase 3 beta
HRP	Horseradish peroxidase
IMC	Integrated modulation contrast (Hoffman contrast)
KEGG	Kyoto Encyclopedia of Genes and Genomes
LC-MS/MS	Liquid chromatography tandem mass spectrometry
LFQ	Label free quantification
MFF	Mitochondrial fission factor
MFN	Mitofusin
MOAS	Mitochondria on a string
MRM	Multiple reaction monitoring
NME3	Nucleoside diphosphate kinase 3
NMOC	Non mitochondrial oxygen consumption
OCR	Oxygen consumption rate
OXPHOS	Oxidative phosphorylation
PBS	Phosphate-buffered saline
PCC	Pearson’s correlation coefficient
PGCs	Polyploid giant cells
PVDF	Polyvinylidene difluoride
ROS	Reactive oxygen species
STRING	Search Tool for the Retrieval of Interacting Genes/Proteins
TEM	Transmission electron microscopy
TNT	Tunneling nanotubes
VDAC	Voltage dependent anion channel
