# Supplementary material for: Mitochondrial Dynamics Participate in an Early Metabolic Adaptation of Glioblastoma Multiforme T98G Cells to Doxorubicin-Induced Chemotherapeutic Stress
Source: Cells. 2026 May 14;15(10):899. doi: 10.3390/cells15100899 (PMC13204552; doi:10.3390/cells15100899)
Supplement: Supplementary file 1 [file cells-15-00899-s001.zip › cells-4237553-supplementary.pdf]

SUPPLEMETARY DATA

Mitochondrial dynamics participate in an early metabolic adaptation of glioblastoma multiforme cells to doxorubicin-induced chemotherapeutic stress

Maciej Pudełek[1,2*], Maksym Pudełek[1], Julia Przeniosło[1], Sylwia Kędracka-Krok[3], Zbigniew Madeja[1] and Jarosław Czyż[1*]
[1]Department of Cell Biology, Faculty of Biochemistry, Biophysics and Biotechnology, Jagiellonian University, Gronostajowa 7, 30-387 Krakow, Poland
[2]Doctoral School of Exact and Natural Sciences, Jagiellonian University, Krakow, Poland
[3]Department of Physical Biochemistry, Faculty of Biochemistry, Biophysics and Biotechnology, Jagiellonian University, Gronostajowa 7, 30-387 Krakow, Poland

Running title: Mitochondrial dynamics and metabolic GBM plasticity
***Correspondence to**: maciej.pudelek@uj.edu.pl and jarek.czyz@uj.edu.pl

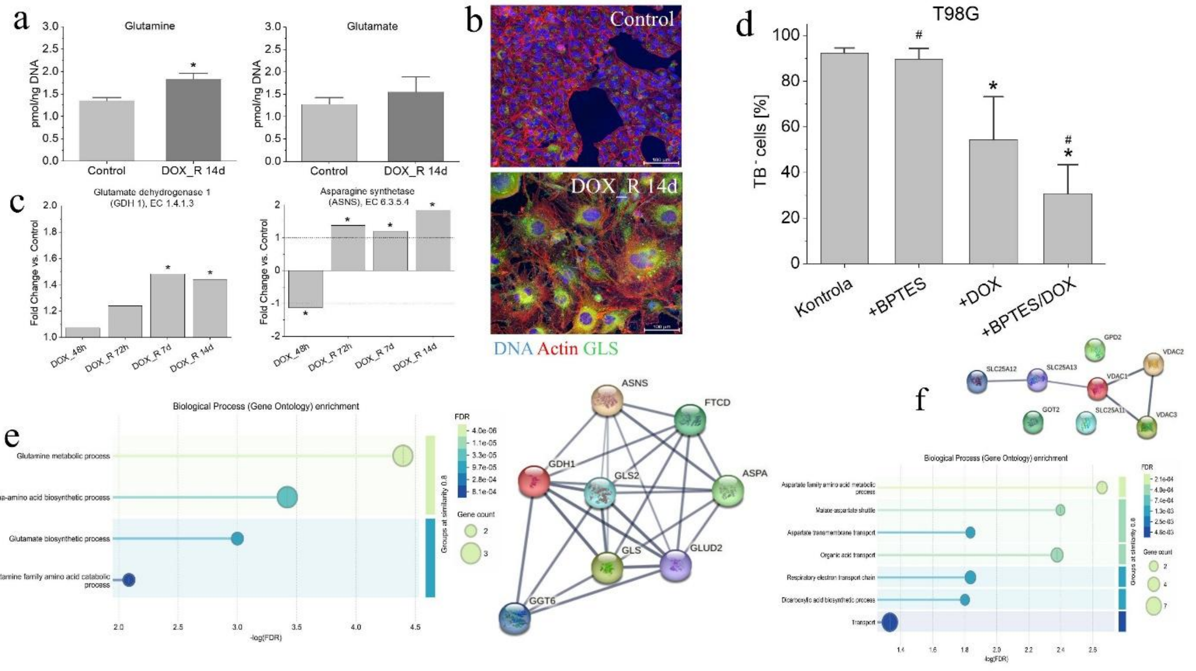

Figure S1. Mobilization of glutaminolytic axis and VDACs in T98G cells under DOX-induced stress. (a) Glutamine and glutamate levels in DOX_R 14d T98G cells estimated with quantitative LC-MS/MS in relation to DNA content. (b) Subcellular distribution of glutaminase (GLS) in DOX_R 14d cells visualized by immunocytochemistry. Scale bars = 100 µm. (c) Levels of glutamate dehydrogenase (GDH) and asparagine synthetase (ASNS) in T98G cells following their pulse DOX treatment (1 mM; 48 h) estimated with LC-MS/MS (fold change>1.2 vs. control). (d) Effect of BPTES (GLS inhibitor; 10 µM) on the DOX (1 mM) sensitivity of T98G cells estimated with trypan blue exclusion assay. (e) Interactome of "glutamine/glutamate metabolism" proteins identified in DOX_R 14d cells using the STRING database. (f) STRING-derived interactomes and functional classification of significantly up-regulated mitochondrial ion transporters identified in DOX_R 14d cells (fold change>1.2). Statistical significance was assessed by non-parametric Mann–Whitney test; *$p < 0.05$ vs. control (a,d) or ANOVA with permutation FDR < 0.05 and Tukey post-hoc ($p < 0.05$ vs. control; c,e,f). Bars represent SD (a,d)). Data representative for 3 independent biological replicates. **Note the mobilization and up-regulation of glutaminolytic enzymes together with enrichment of VDAC-associated mitochondrial transporters in DOX_R 14d T98G cells.**

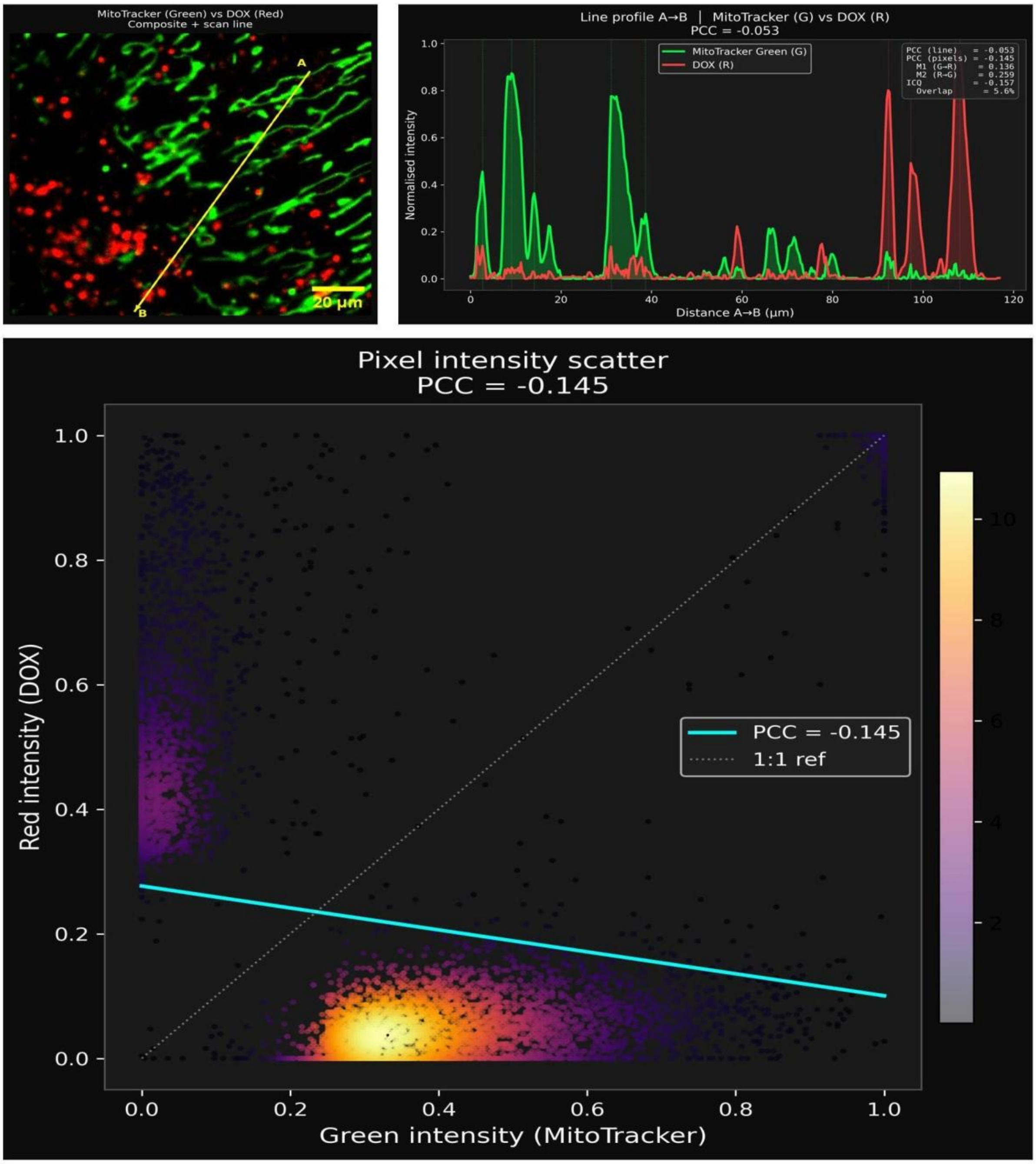

Figure S2. Experimental approach for the analysis of DOX (red) and MitoTracker Green signals in T98G_DOX14d cells (cf. Fig. 1e). A representative cell image was analyzed along the indicated line (yellow; upper panel) using a Claude Sonnet 4.6 Extended algorithm. The lower panel shows the distribution of fluorescence intensities in the red (DOX) and green (MitoTracker) channels, used to calculate the Pearson correlation coefficient (PCC). The image is representative of >50 cells from three independent biological replicates. Scale bar = 20 µm. **Note the low PCC values, indicating extra-mitochondrial sequestration of DOX.**

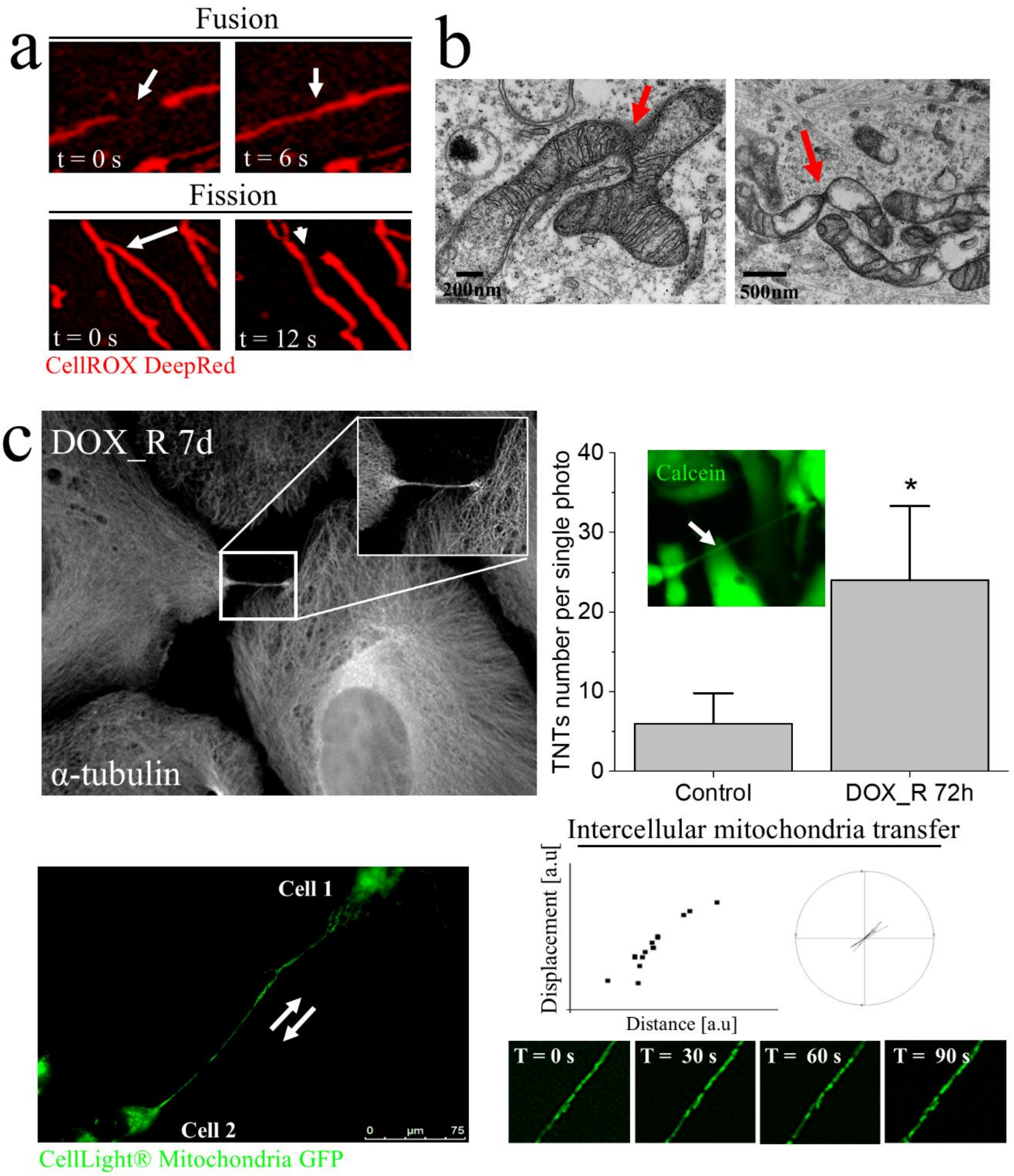

Figure S3. Mitochondrial dynamics in pulse DOX-treated PGCs. (a) Mitochondrial fusion and fission events in mitochondrial networks of pulse DOX-treated PGCs (DOX_R 14d) visualized by live time-lapse imaging of CellROX™ Deep Red-stained cells. (b) Representative transmission electron micrographs (TEM) of fusion/fission loci in the mitochondrion of DOX_R 14d cells (arrows). (c) Intercellular tunneling nanotubes (TNTs) between T98G cells at 7th day after their pulse DOX treatment (1 mM; 48h) visualized with a-tubulin (left) and calcein staining (right). Lower panel shows mitochondria within TNTs (CellLight® Mitochondria GFP; left) and kinetics of their TNT-mediated transfer (right). Plots depict changes of their positions along the nanotube axis. Scale bars = 200 nm and 500 nm (b) and 75 µm (c). Data representative for 3 independent biological replicates. **Note the nanotube-mediated mitochondrial transfer and fusion/fission events during T98G adaptation to DOX.**

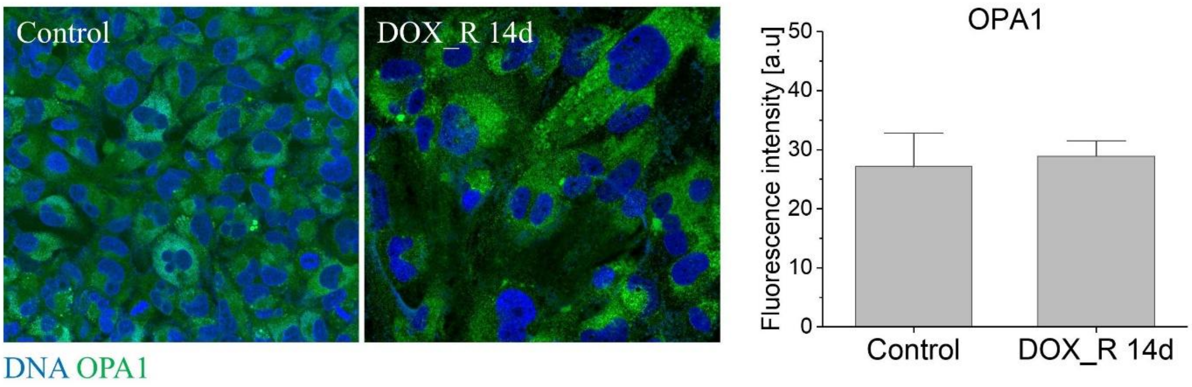

Figure S4. Effect of pulse DOX treatment (1 µM; 48 h) on OPA1 levels on the 14th day after DOX removal. Cells were analyzed with quantitative fluorimetric approach at 14th day after DOX removal. Scale bars = 50 µm. Statistical significance was assessed by non-parametric Mann–Whitney test; *p < 0.05 vs. control. Bars represent SD. Data representative for 3 independent biological replicates. **Note the lack of differences in OPA1 levels between control and pulse DOX-treated T98G variant.**

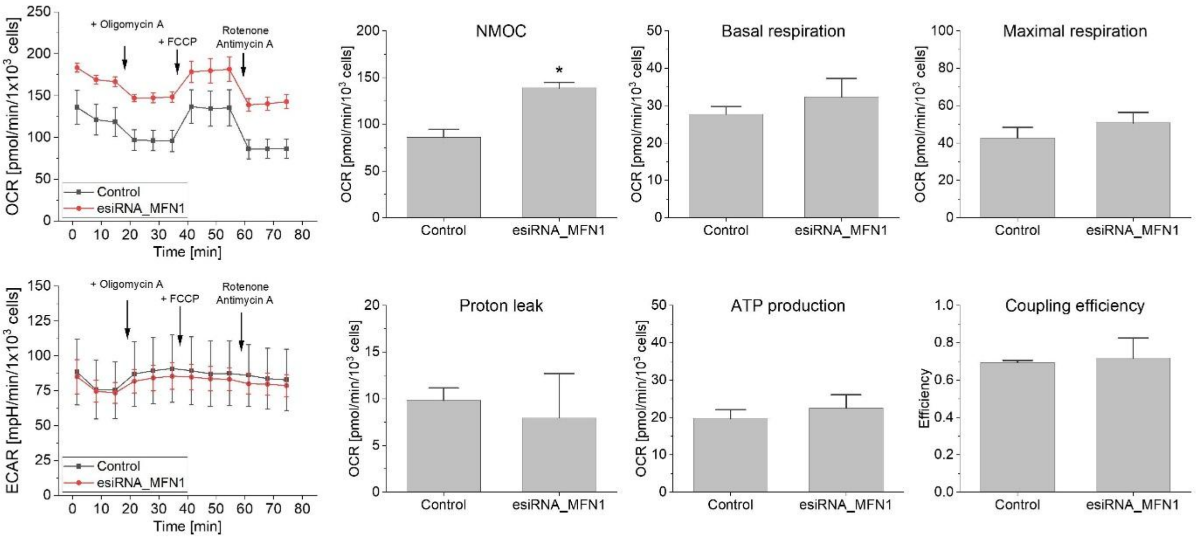

Figure S5. The effect of MFN1 down-regulation by MFN1esiRNA on the parameters of mitochondrial respiration estimated with Seahorse XFp (MitoStress assay) 48 h after the transfection. Data from 3 independent biological replicates. Statistical significance was assessed by non-parametric Mann–Whitney test; *$p < 0.05$ vs. control. Bars represent SD. **Note the lack of MFN1 effects on mitochondrial respiration.**

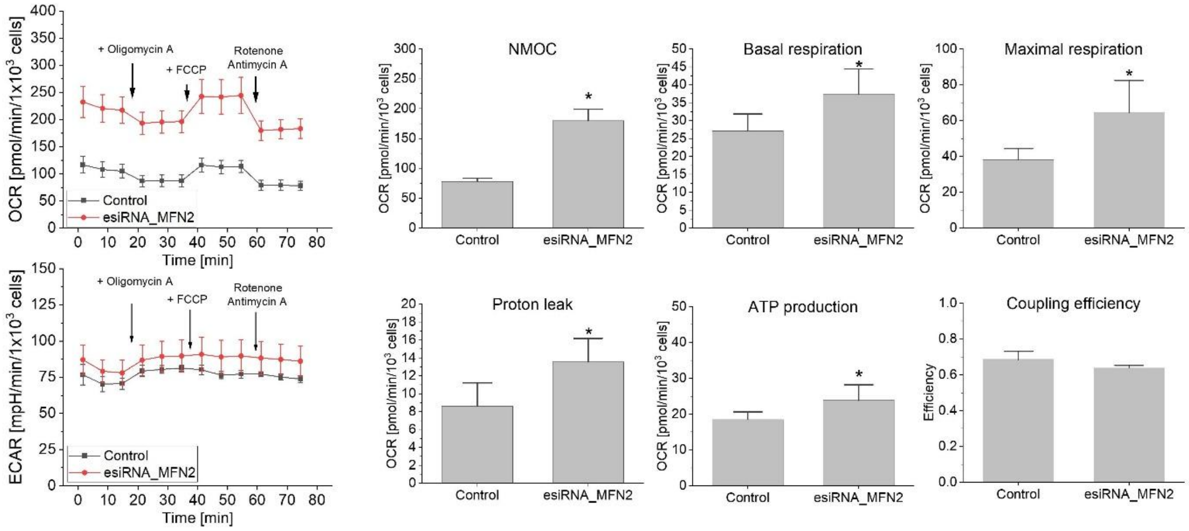

Figure S6. The effect of MFN2 down-regulation by MFN2esiRNA on the parameters of mitochondrial respiration estimated with Seahorse XFp (MitoStress assay) 48h after the transfection. Data from 3 independent biological replicates. Statistical significance was assessed by non-parametric Mann–Whitney test; *p < 0.05 vs. control. Bars represent SD. **Note the increased mitochondrial respiration following MFN2 down-regulation.**

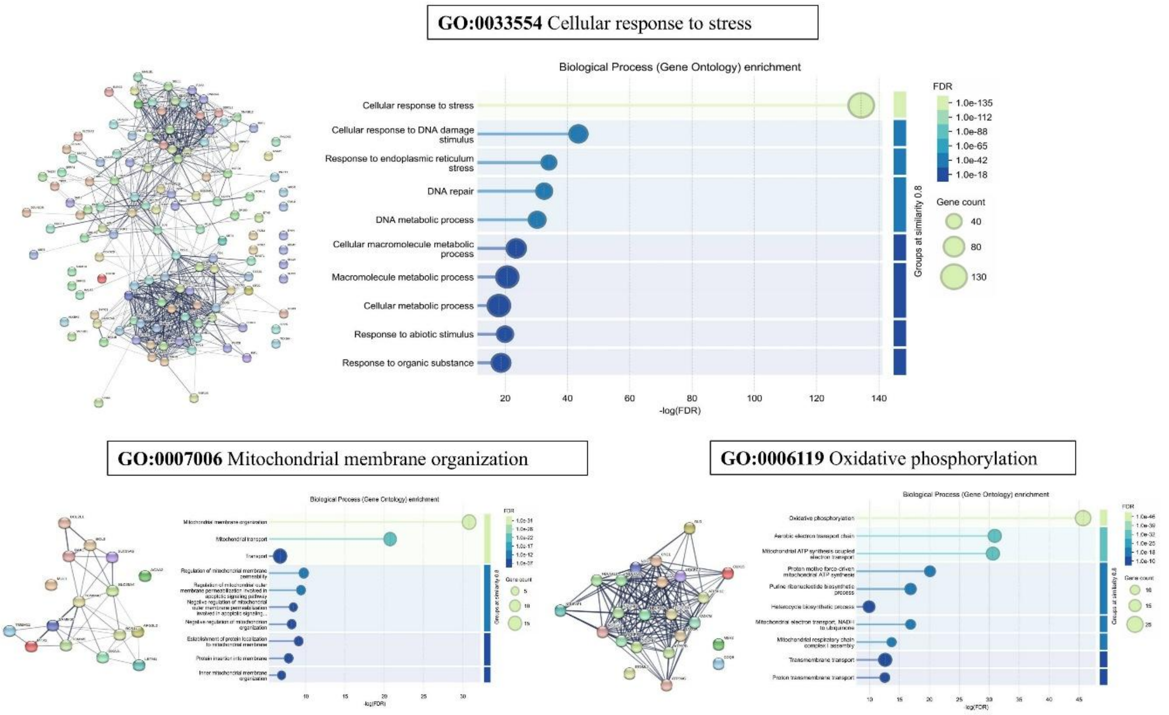

Figure S7. Interactomes of (i) „cellular response to stress", (ii) „mitochondrial membrane organization" and (iii) „oxidative phosphorylation" proteins significantly up-regulated in T98G cells undergoing MFN2 down-regulation and quantified by LC–MS/MS. Proteins with fold change >1.2 were classified into functional clusters in STRING database according to Gene Ontology. Statistical significance was assessed by ANOVA with permutation FDR < 0.05 and Tukey post-hoc ($p<0.05$ vs. control). Data representative for 3 independent biological replicates. **Note the strong engagement of compensatory mechanisms encompassing not only the organization of these organelles but also their bioenergetic performance and the cellular stress-response processes.**

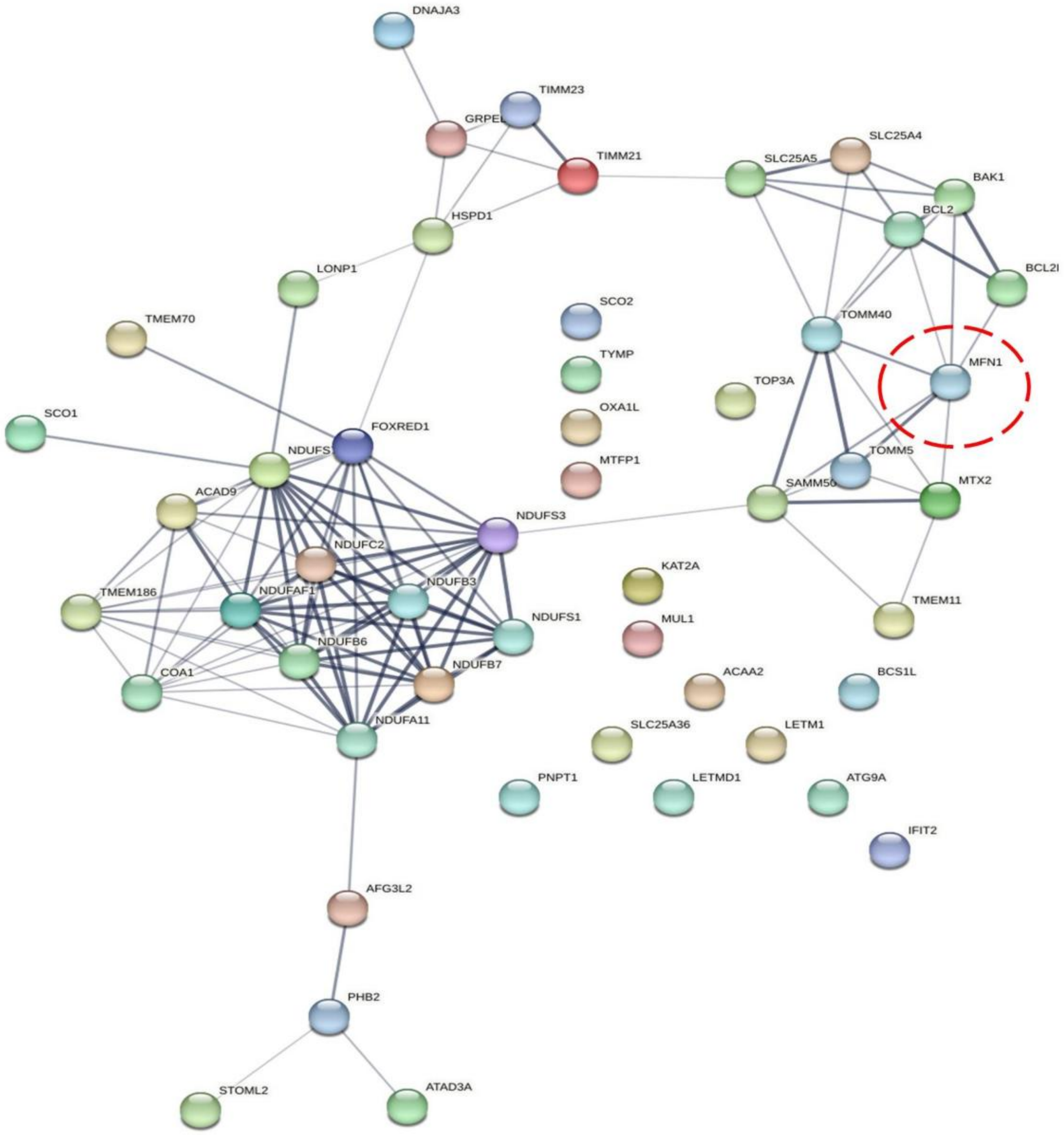

Figure S8. Interactome of proteins involved in „mitochondria organization", which were significantly up-regulated in T98G cells undergoing MFN2 down-regulation and quantified by LC–MS/MS. Proteins with fold change >1.2 were classified into functional clusters in STRING database according to Gene Ontology. Statistical significance was assessed by ANOVA with permutation FDR < 0.05 and Tukey post-hoc (p<0.05 vs. control). Data representative for 3 independent biological replicates. **Note the increased level of MFN1, which is likely part of a compensatory response to the MFN2 down-regulation.**

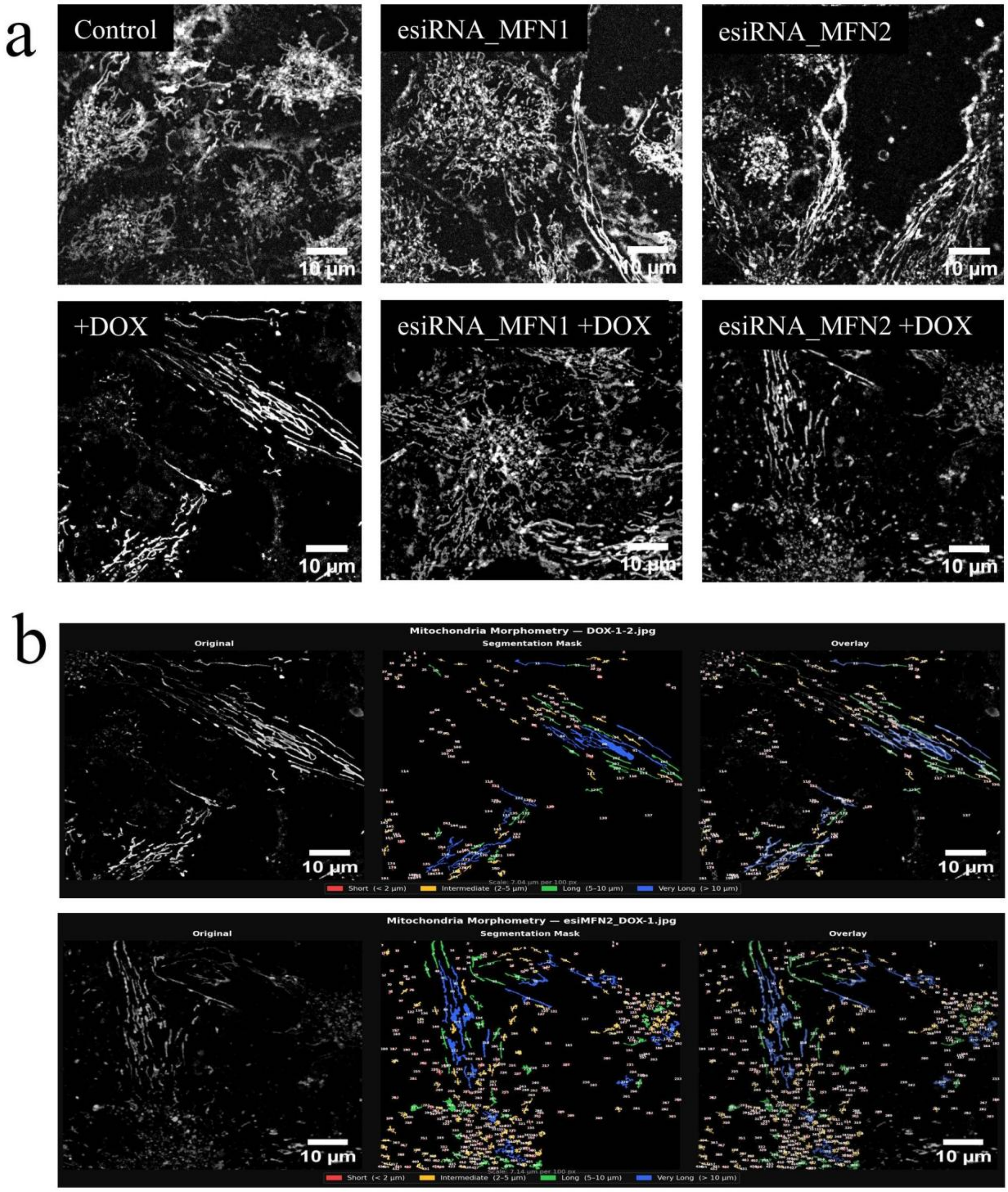

Figure S9. Analytic approach towards the estimation of the effect of MFN1 or MFN2 down-regulation on the morphology of T98G mitochondria following pulse DOX treatment. (a) Representative images of MitoTracker Green –stained cells. (b) Segmentation of T98G mitochondria performed with Python Claude Pro Sonnet 4.6 Extended algorithm. Images representative for >50 cells from 3 independent experimental replicates. Scale bars = 10 µm.

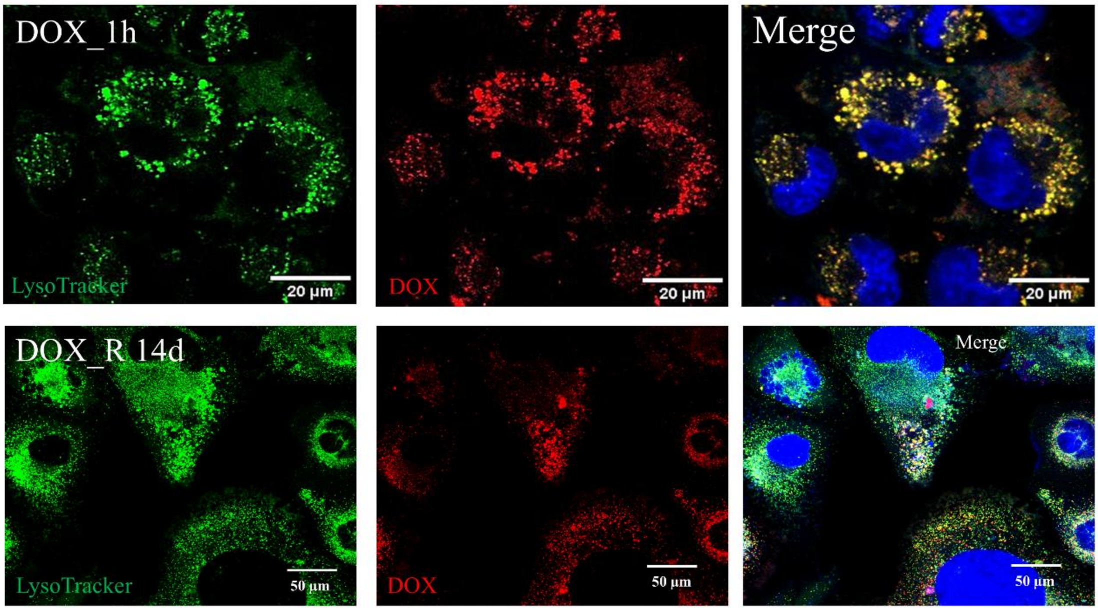

Figure S10. Dynamics of DOX sequestration in T98G cells. T98G cells were pulse DOX treated and stained with LysoTracker (green)/DNA (blue) 1h and 14 days thereafter to visualize the intracellular localization of DOX. Note the lysosomal DOX accumulation observed immediately after DOX application and its further persistence, accompanied by the appearance of DOX$^-$ lysosomes. Scale bars 20 and 50 µm. Data representative for 3 independent biological replicates.

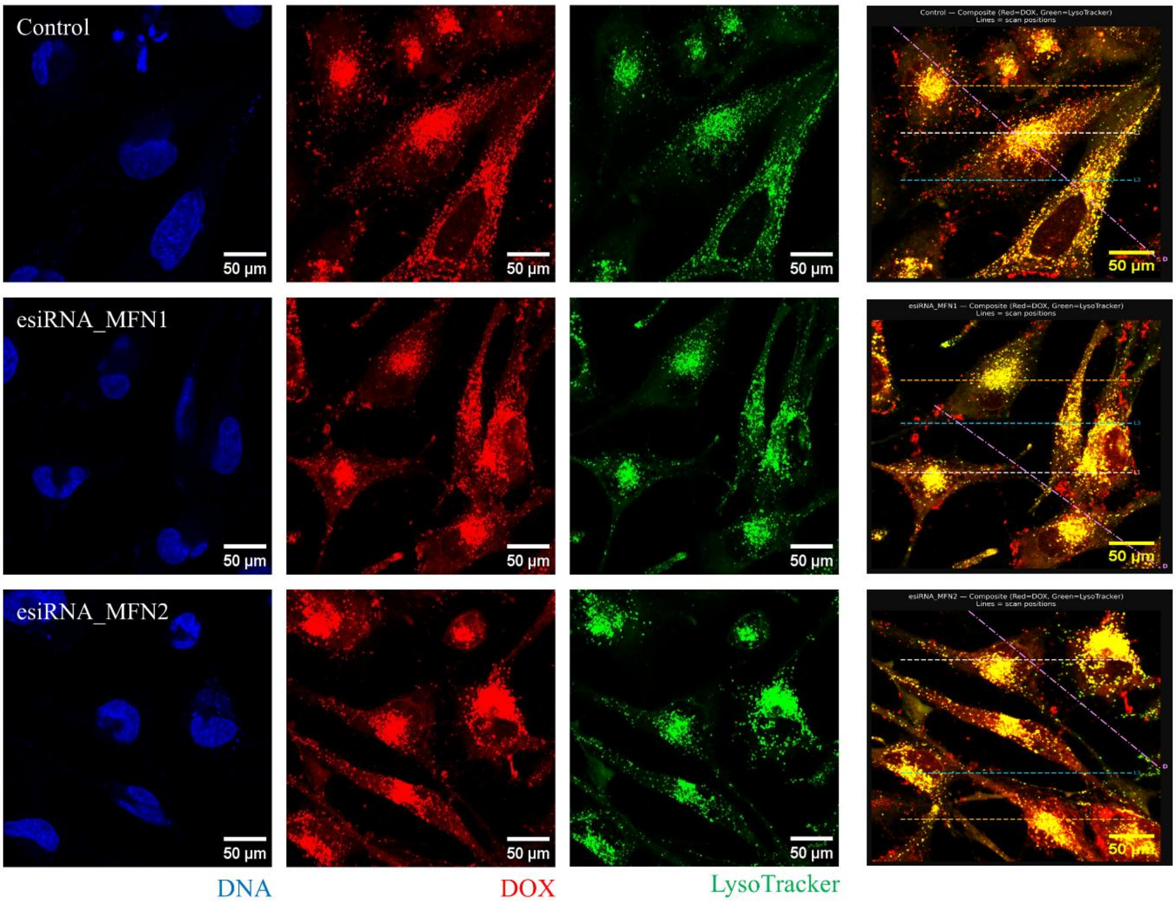

Figure S11. Experimental approach for the analysis of the co-localization of DOX (red) and LysoTracker signals in T98G cells undergoing MFN1 or MFN2 silencing. Images were analyzed along the indicated lines using a Claude Sonnet 4.6 Extended algorithm (cf. Fig. S12). **Scale bars = 50 µm.** A representative cell image was analyzed along the indicated lines (yellow; right collumn) using a Claude Sonnet 4.6 Extended algorithm to draw the distribution of fluorescence intensities in the red (DOX) and green (LysoTracker) channels, used to calculate the Pearson correlation coefficient (PCC; cf. Fig. S12). The image is representative of >50 cells from three independent biological replicates. Scale bars = 50 µm.

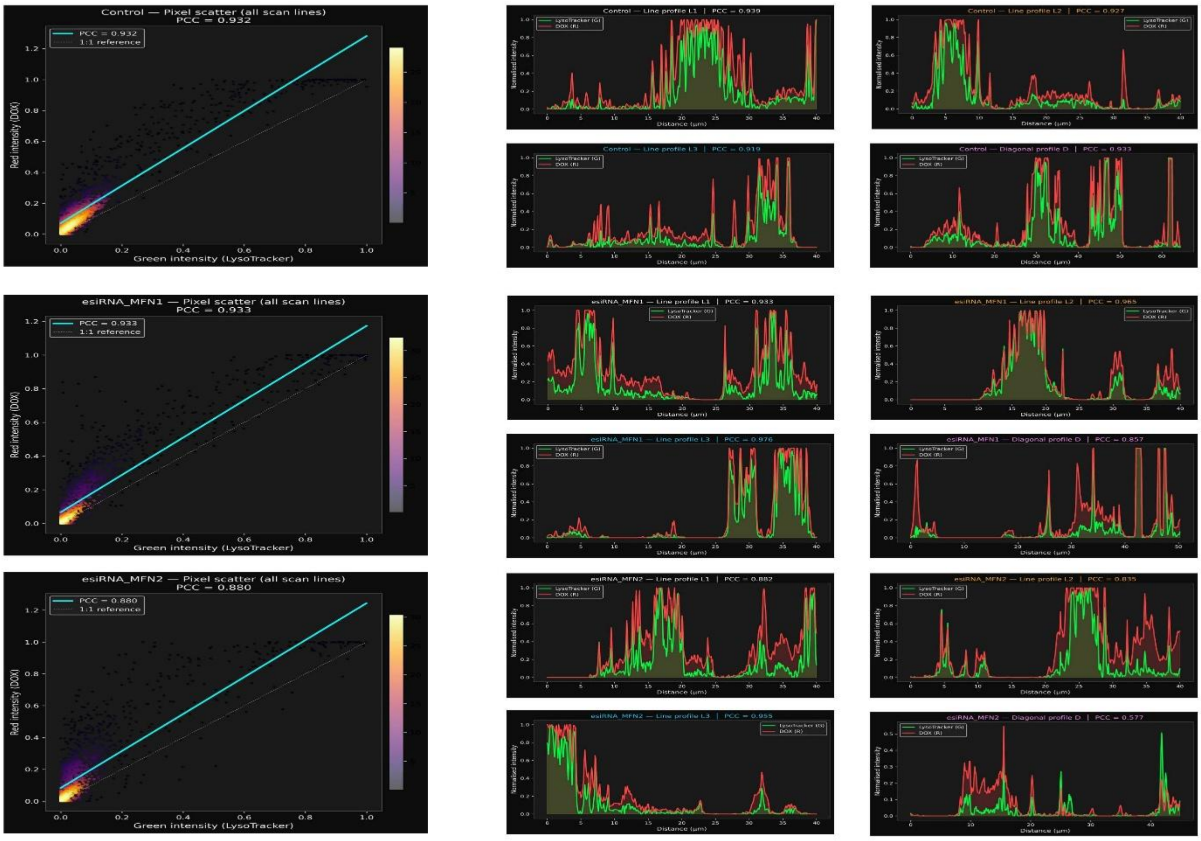

Figure S12. Experimental approach for the analysis of the co-localization of DOX (red) and LysoTracker signals in T98G cells (control; upper row) undergoing MFN1 (middle) or MFN2 silencing (lower row). Histograms show distribution of fluorescence intensities in the red (DOX) and green (LysoTracker) channels, used to calculate the Pearson correlation coefficient (PCC) with a Claude Sonnet 4.6 Extended algorithm. Data representative of >50 cells from three independent biological replicates. **Note the high PCC values, indicating on lysosomal DOX retention in T98G cells.**

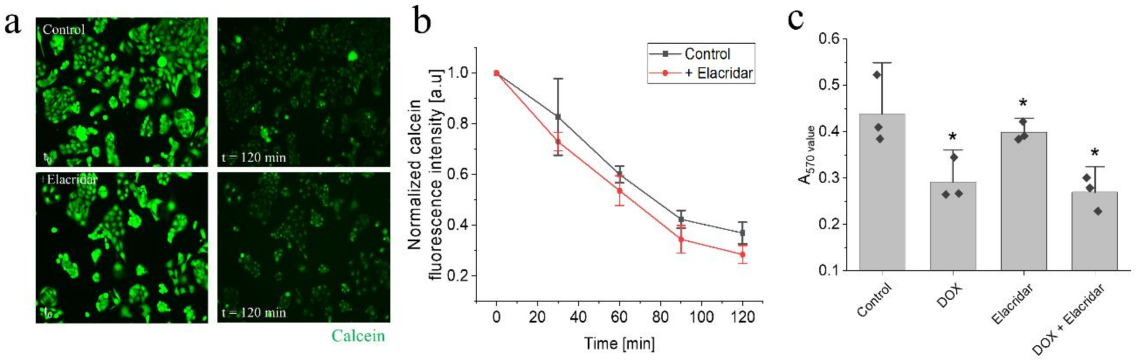

Figure S13. Redundancy of the DOX relocation system in T98G cells. (a-b) Effect of pharmacological ABCB1 inhibition on the intensity of calcein efflux and (c) DOX cytotoxicity in T98G populations measured by MTT test. Scale bars = 100 μm. Statistical significance was assessed by non-parametric Mann–Whitney test; *p < 0.05 vs. control (n = 3 independent biological replicates). **Note that pharmacological perturbation was not related to detectable change in T98G DOX sensitivity of T98G cells.**

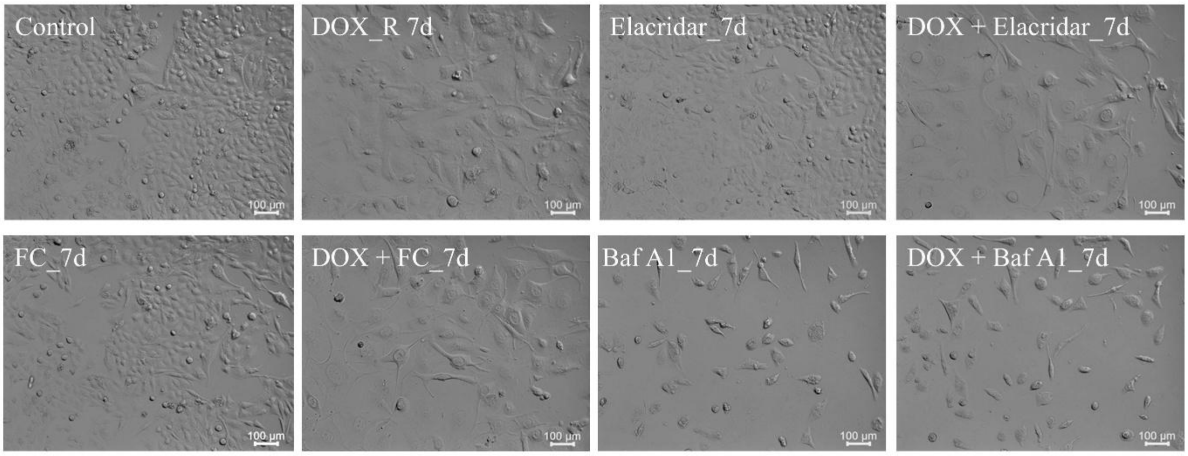

Figure S14. Images of the effect of chemical ABCB1 and V-type proton ATPase inhibitors on the morphology of T98G cells under DOX-induced stress. Data representative for 3 independent biological replicates. Scale bars = 100 µm.
